# Advancements in polyol synthesis: expanding chemical horizons and Néel temperature tuning of CoO nanoparticles

**DOI:** 10.1038/s41598-024-54892-2

**Published:** 2024-05-31

**Authors:** Miran Baričić, Jorge M. Nuñez, Myriam H. Aguirre, David Hrabovsky, Mahamadou Seydou, Carlo Meneghini, Davide Peddis, Souad Ammar

**Affiliations:** 1 ITODYS, UMR CNRS 7086, Université Paris Cité, 15 Rue de Jean Antoine de Baif, 75013 Paris, France; 2 Istituto di Struttura della Materia, ISM-CNR, 00015 Monterotondo Scalo, Rome, Italy; 3 Dipartimento di Scienza, Università degli Studi Roma Tre, Via della Vasca Navale, 84-00146 Rome, Italy; 4 Instituto de Nanociencia y Nanotecnologìa, CNEA, CONICET, S. C., Bariloche, 8400 Rio Negro, Argentina; 5 Instituto Balseiro (UNCuyo, CNEA), Av. Bustillo 9500, S. C. de Bariloche 8400, Rio Negro, Argentina; 6 Instituto de Nanociencias y Materiales de Aragón-CSIC-Universidad de Zaragoza, Mariano Esquillor S/N, 50018 Zaragoza, Spain; 7 Laboratorio de Microscopías Avanzadas, Universidad de Zaragoza, Mariano Esquillor S/N, 50018 Zaragoza, Spain; 8 Dept. Física de La Materia Condensada, Universidad de Zaragoza, C/ Mariano Esquillor S/N, Zaragoza, Spain; 9 IMPMC, UMR CNRS 7590, Sorbonne Université, 6 Place Jussieu, 75005 Paris, France; 10 Università degli Studi di Genova, Dipartimento di Chimica e Chimica Industriale, Via Dodecaneso 31, 16146 Genova, Italy

**Keywords:** Chemistry, Materials science, Nanoscience and technology, Physics

## Abstract

The polyol synthesis of CoO nanoparticles (NPs) is typically conducted by dissolving and heating cobalt acetate tetrahydrate and water in diethylene glycol (DEG). This process yields aggregates of approximately 100 nm made of partially aligned primary crystals. However, the synthesis demands careful temperature control to allow the nucleation of CoO while simultaneously preventing reduction, caused by the activity of DEG. This restriction hinders the flexibility to freely adjust synthesis conditions, impeding the ability to obtain particles with varied morpho-structural properties, which, in turn, directly impact chemical and physical attributes. In this context, the growth of CoO NPs in polyol was studied focusing on the effect of the polyol chain length and the synthesis temperature at two different water/cations ratios. During this investigation, we found that longer polyol chains remove the previous limits of the method, allowing the tuning of aggregate size (20–150 nm), shape (spherical-octahedral), and crystalline length (8–35 nm). Regarding the characterization, our focus revolved around investigating the magnetic properties inherent in the synthesized products. From this point of view, two pivotal findings emerged. Firstly, we identified small quantities of a layered hydroxide ferromagnetic intermediate, which acted as interference in our measurements. This intermediate exhibited magnetic properties consistent with features observed in other publications on CoO produced in systems compatible with the intermediate formation. Optimal synthetic conditions that prevent the impurity from forming were found. This resolution clarifies several ambiguities existing in literature about CoO low-temperature magnetic behavior. Secondly, a regular relationship of the NPs' T_N_ with their crystallite size was found, allowing us to regulate T_N_ over ~ 80 K. For the first time, a branching was found in this structure-dependent magnetic feature, with samples of spheroidal morphology consistently having lower magnetic temperatures, when compared to samples with faceted/octahedral shape, providing compelling evidence for a novel physical parameter influencing the T_N_ of a material. These two findings contribute to the understanding of the fundamental properties of CoO and antiferromagnetic materials.

## Introduction

**Figure 1:**
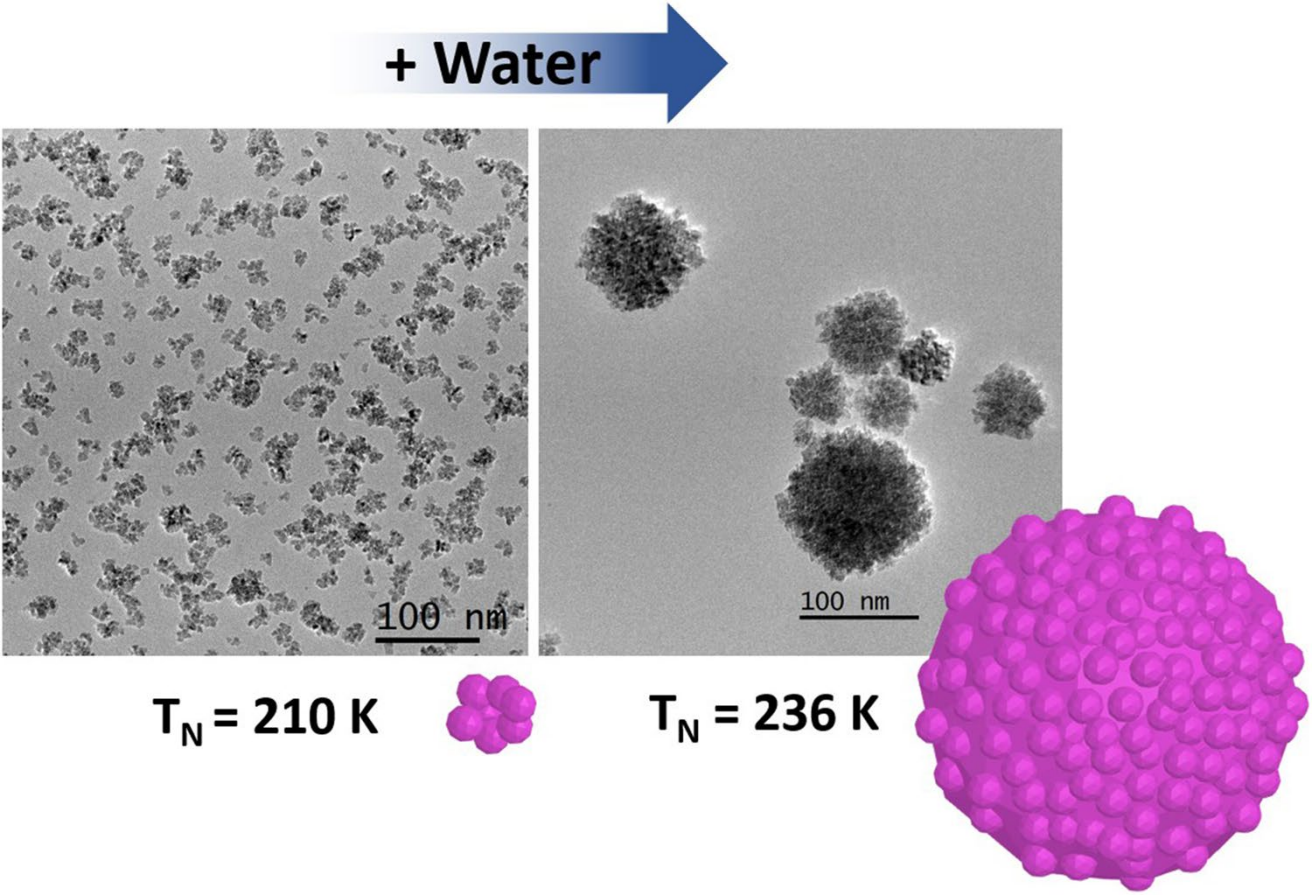
A schematic representation of the effect of water (hydrolysis ratio) on the morphology and Néel temperature of CoO nanostructures (TTEG1 and TTEG2 in the picture).

The polyol synthesis, introduced in 1989 by Fiévet, Lagier and Figlarz^1–4^ is a colloidal chemistry method for the synthesis nanoparticles (NPs). The approach consists in the use of polyols (i.e., organic molecules with multiple –OH groups) as solvents due to their key properties. For example, their high polarity and boiling point make them similar to water, especially for the capacity to dissolve metal salts, but without the inconvenience of a low boiling temperature, often demanding for the use of an autoclave. Other advantages consist in the chelating properties of the polyols, the increased viscosity—which helps a diffusion-controlled particles’ growth and makes the morphology more controllable—and reducing properties. These features served well the initial objective of producing metal particles and is still useful for controlling the oxidation state of a material^3–5^. In the context of CoO, the material is not the thermodynamically favored cobalt oxide at room temperature in the Co–O system^6,7^, where CoO is stable just above 900 °C, and can be achieved with a reducing atmosphere^8^. In the case of wet chemistry, similar considerations are valid in the Co–H_2_O system^9^, where again CoO is not thermodynamically favored at any pH or potential, evidencing the impossibility of producing CoO directly in water. This calls for the development of specific conditions allowing the formation of CoO instead of Co_3_O_4_, or other oxides/hydroxides. Such conditions can be met by the forced hydrolysis polyol method. Indeed, many in-solution synthesis approaches have been successfully employed to prepare cobalt oxide nanoparticles, such as microwave-assisted precipitation^10^, hydrothermal^11^, solvothermal^12^, chemical bath deposition^13^ among others, but in all these cases, only Co_3_O_4_ nanoparticles were obtained. Co_3_O_4_/CoO nanoparticles were produced using for instance self-combustion route^14^. Co/CoO were also successfully prepared using organometallic thermal decomposition in non-polar solvent, in presence of Na(AOT) (sodium bis(2-ethylhexyl) sulfosuccinate)^15^ but it remains difficult to produce directly pure CoO nanoparticles in solution. The requirement was met with the forced hydrolysis in polyol media, as shown by Poul et al*.*^16^, which extended the polyol synthesis to CoO by dissolving tetrahydrate cobalt acetate (Co(ac)_2_·4H_2_O) in polyol and in presence of a given amount of water, usually expressed as the hydrolysis ratio ($h=\left[{{\text{H}}}_{2}{\text{O}}\right]/\left[{{\text{Co}}}^{2+}\right]$). Increasing the basicity of the reaction medium should also favor Co_3_O_4_ production, but in polyols, the addition of hydroxide anions usually promotes reduction reactions and the production of Co metal^4^. In fact it is easier to produce CoO by forced hydrolysis in polyols than Co_3_O_4_^3,16^, which, conversely, is easily obtained in water- and ethanol-based solutions^10–13,17,18^. CoO polyol synthesis is typically performed in diethylene glycol (DEG)^16,19–24^, and it is known to form layered hydroxide salt (LHS) structures prior to CoO precipitation^19,25^. The temperature needs careful regulation at ~ 180 °C, since lower temperatures do not trigger the CoO nucleation, and higher ones provoke reduction reactions forming high quantities of metallic Co and Co carbides. In the right conditions, the method provides spherical aggregates of ~ 100 nm made of ~ 5 nm primary CoO particles. Interestingly, the primary particles do not aggregate in random directions, but a certain degree of crystalline alignment was observed^21^. Different explanations can be given to the phenomenon: theoretical simulations^21^ suggest that the result might be due to oriented aggregation^26,27^ mediated by polyol-polyol interactions. At the same time, kinetic studies assisted by transmission electron microscopy observations performed on other oxides (such as the one of Cannas et al*.*^28^) showed that NPs aggregates can nucleate on layered intermediates such as β-Co(OH)_2_, and suggested that this mediated nucleation step might induce the oriented growth. However, to the best of our knowledge, polyol syntheses of CoO have continued to be performed in DEG^16,20–24^. No studies have been made to investigate systematically the effect of the polyol solvent’s chemical nature on the production of CoO in polyol environment, and the role of hydrolysis ratio remains unexplored. Regarding the magnetic aspects, cobalt(II) oxide is an antiferromagnetic (AFM) material with a ~ 290 K Néel temperature (T_N_)^29,30^ and a face centered cubic (fcc) rock-salt structure. Besides being considered an interesting case of study for basic magnetism science^31–34^, large applicative interest is found on nano-crystallized CoO in the fields of water splitting^35–37^, alcohol reforming^36,38,39^ and lithium ion battery energy storage^38,40–42^. Focusing on nano-sized CoO magnetic studies, literature provides studies on several magnetic aspects of the material, but nevertheless conflicting properties are reported. For instance, it is not yet clarified the magnitude of the surface effect on the total magnetic properties at the nanoscale. Co^2+^ surface atoms were shown to have a stronger spin–orbit coupling^43^, and uncompensated surface magnetic moments or anion vacancies were considered to explain observations such as weak ferromagnetic (FM) phenomena, irreversible magnetism or paramagnetic contributions to the total magnetization^33,44–52^. AFM materials syntheses are also susceptible to the formation of other magnetic materials that can make the landscape more complex. This will be shown to be particularly relevant in the case of polyol synthesis (and possibly other ones) because of the formation of layered hydroxide structures with ferromagnetic behavior at low temperatures^25,53^. In the light of this information, it is not surprising that the term “anomalous” is recurrent in describing some CoO properties^33,45,47,54^. Starting from this fascinating landscape, our study investigates the effect of some specific synthesis parameters such as the temperature and the polyol length at two different hydrolysis ratios (experimental scheme in Figure S1). As a result, the feasibility conditions of the synthesis were significantly expanded, and new morphologies were produced at both hierarchical levels (i.e., primary crystals and aggregates). Furthermore, water was found to influence the NPs crystal size and aggregation size, also influencing the magnetic properties (Fig. 1). The magnetic properties have been also investigated focusing on both size dependence of T_N_ and role of impurities in dominating the magnetic behavior of nanostructured CoO. Finally, a possible new physical parameter, apart from size, was found to influence T_N_.

## Methods

### Synthesis

All the samples were synthesized by means of the polyol method. All the chemicals were purchased from Sigma-Aldrich. Cobalt acetate tetrahydrate (Co(ac)_2_·4H_2_O) was dissolved in di-, tri-, tetra- or polyethylene glycol (DEG, TEG, TTEG and 400 g mol^−1^ PEG, corresponding to ~ 9 EG units) in presence of deionized water in a three necked flask and stirred with a mechanical stirrer at 450 rpm. The lateral necks are used for a thermocouple and a waterless air condenser (~ 42 cm of effective length). The mixture is left to stir for 20–30 min, and then heated until the chosen temperature at a 6 °C/min rate. After 18 h, the system is cooled at room temperature by removing the heat source; the product is washed three times with ethanol and centrifuged each time for 15 min at 10,000 rpm. Further details can be found in SI (Tables S2 and S3).

For each of the polyols used, two nominal hydrolysis ratios ($h=\left[{{\text{H}}}_{2}{\text{O}}\right]/\left[{{\text{Co}}}^{2+}\right]$, 7 and 46) and three temperatures are employed (180 °C, 205 °C and 235 °C), for a total of 24 syntheses. The samples were named after the polyol used (DEG, TEG, TTEG or PEG), the hydrolysis ratio (“*a”* for *h* = 7 and “*b”* for *h* = 46) and the temperature chosen (180, 205 and 235 °C).

### Instrumental characterization

X-ray powder diffraction (XRPD) patterns were measured on the samples’ powders with a PANalytical X’pert Pro working in Bragg–Brentano θ–θ reflection geometry (multichannel X’celerator detector, Co Kα X-ray tube, 40 kV, 40 mA). The NPs’ sizes were evaluated by means of Rietveld refinement, performed with the software MAUD^55^. Transmission electron microscopy (TEM) pictures in low magnification were obtained using a Jeol 2100 Plus (200 kV, LaB_6_ source, EDS Si(Li) detector, CCD GATAN Multiscan Camera). TEM pictures were elaborated with the software ImageJ to assess the statistical distribution of the particles’ sizes, counting approximately 400 particles for each sample. Feret diameter (i.e., the maximum caliper diameter)^56^ is used to measure the size of particles.

High resolution microscopy (HR-TEM) was performed in a FEI Titan 80–300 keV with a spherical aberration corrector at the objective lens. High-angle annular dark-field scanning transmission electron microscopy (HAADF-STEM) images were taken in a FEI Titan 80–300 keV equipped with a CESCOR Cs-probe corrector from CEOS Company and monochromator (Potential 3000, Excitation 0.7). For Electron Energy Loss Spectroscopy (EELS) and Energy Loss Near Edge Structure (ELNES) experiments, the microscope is fitted with a Gatan Energy Filter Tridiem 866 ERS and used simultaneously with the monochromator (Experimental condition: detector DF4, Camera length 48 mm, Mask 2.5 mm, dispersion 0.15 eV/pixel, C3 30 mm spot size 16). The combination of EELS with STEM mode permits to acquire chemical composition maps and profiles with atomic resolution. Thermogravimetric analysis (TGA) was performed on a Setaram Labsys Evo 1600 machine in an alumina crucible. Fourier-transform infrared (FTIR) spectroscopy was performed in transmission mode on solid powders (KBr method) using a Perkin-Elmer 1750 spectrophotometer between 4000 and 400 cm^−1^ with a resolution of 4 cm^−1^ (at least 20 scans). Ultraviolet–visible–near infrared (UV–Vis–NIR) spectroscopy was performed on a the as-produced powders using a Perkin Elmer-Lambda 1050 spectrophotometer equipped with a polytetrafluoroethylene (PTFE) coated integration sphere. UV–Vis–NIR diffuse reflectance spectra were recorded in the 200–2500 nm wavelength range and analyzed with emphasis on the d–d transitions of 3d^7^ Co^2+^ cations. The magnetic measurements were performed on fresh dry powder samples with a Superconducting Quantum Interference Device (SQUID) magnetometer, on an MPMS XL 7T by Quantum Design. The samples were magnetically studied by means of isothermal magnetization plots (MVsH) at 2.5 and 300 K and by following the thermal variation of the magnetization (MVsT) by means of the zero-field cooled/field cooled (ZFC/FC) protocols. ZFC measurements are conducted by cooling the sample to a low temperature in the absence of magnetic field (H), and then recording the variation of magnetization during heating after the application of a small field (specifically, 2.5 mT); the FC measurement is similar, with the only difference being the application of 2.5 mT since before the cooling process.

## Results and discussion

### Structure and morphology

**Figure 2:**
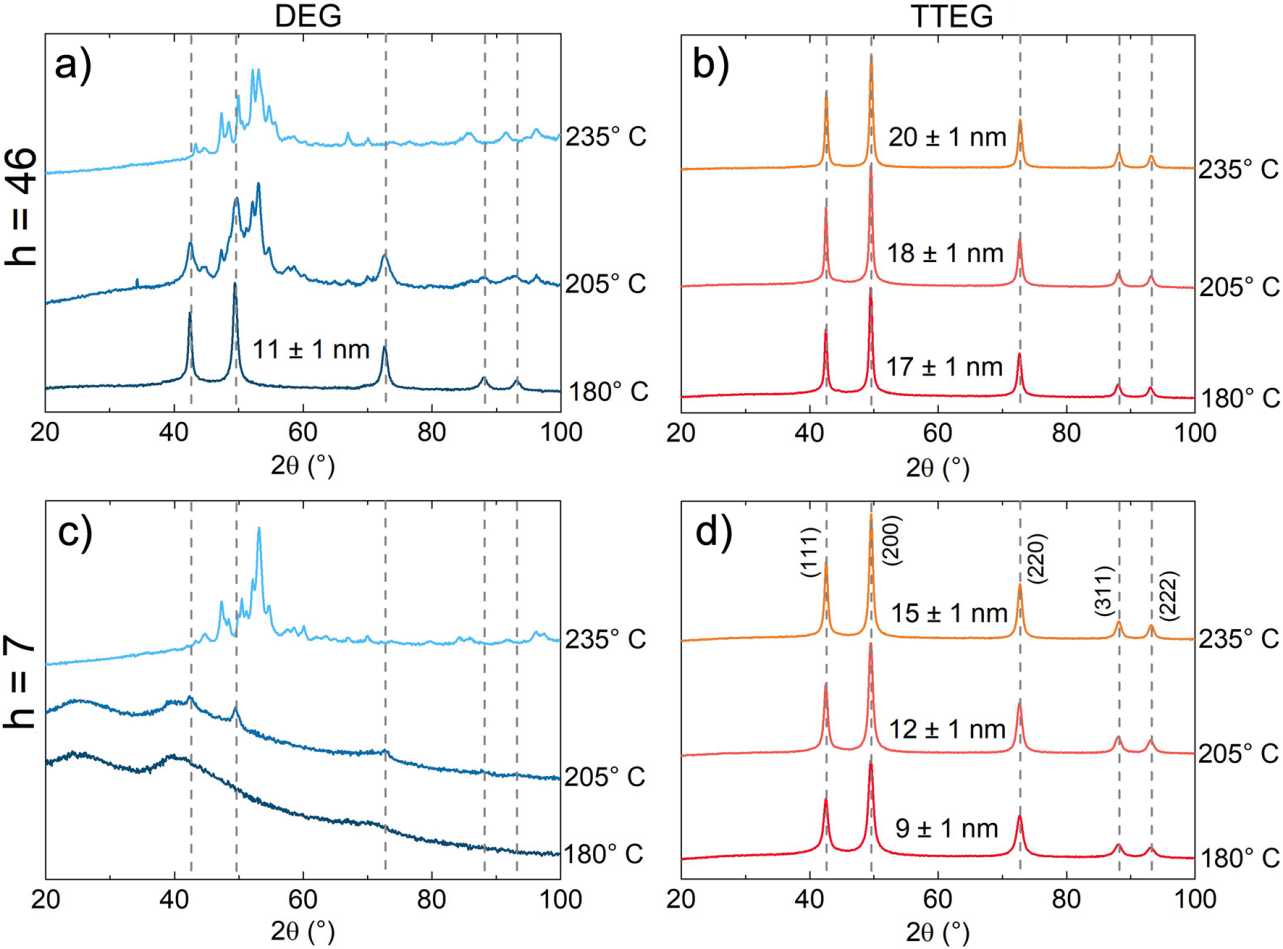
XRPD patterns of all the synthesis tried with DEG (blue) and TTEG (orange) at h = 46 (**a** and **b**) and h = 7 (**c** and **d**).

Twenty-four samples were prepared as summarized in Table S3 and Figure S1. The synthesis conditions typically found in literature for the polyol method are the ones for DEG-b180. The morpho-structural features of the sample are similar to the ones obtained in the work of Gaudisson et al*.*^21^, with ~ 100 nm aggregates made of ~ 4 nm primary particles, and a XRPD crystalline size of ~ 11 nm (Figs. 2, and 4a,b).

From Figure S2, summarizing the XRPD patterns of all the syntheses, clear influences of the investigated parameters can be found. As expected, in presence of DEG, an increase of temperature leads to the massive formation of impurities, such as metallic Co (visible from the reflective layer formed on the bottom of the flask) and a mixture of Co_2_C and Co_3_C, already observed in other studies employing the polyol synthesis^57–59^. At *h* = *7*, just faint CoO peaks are visible. The samples had the appearance of a black lightweight powder, identified as amorphous carbon^60^ (Fig. 2).

**Figure 3:**
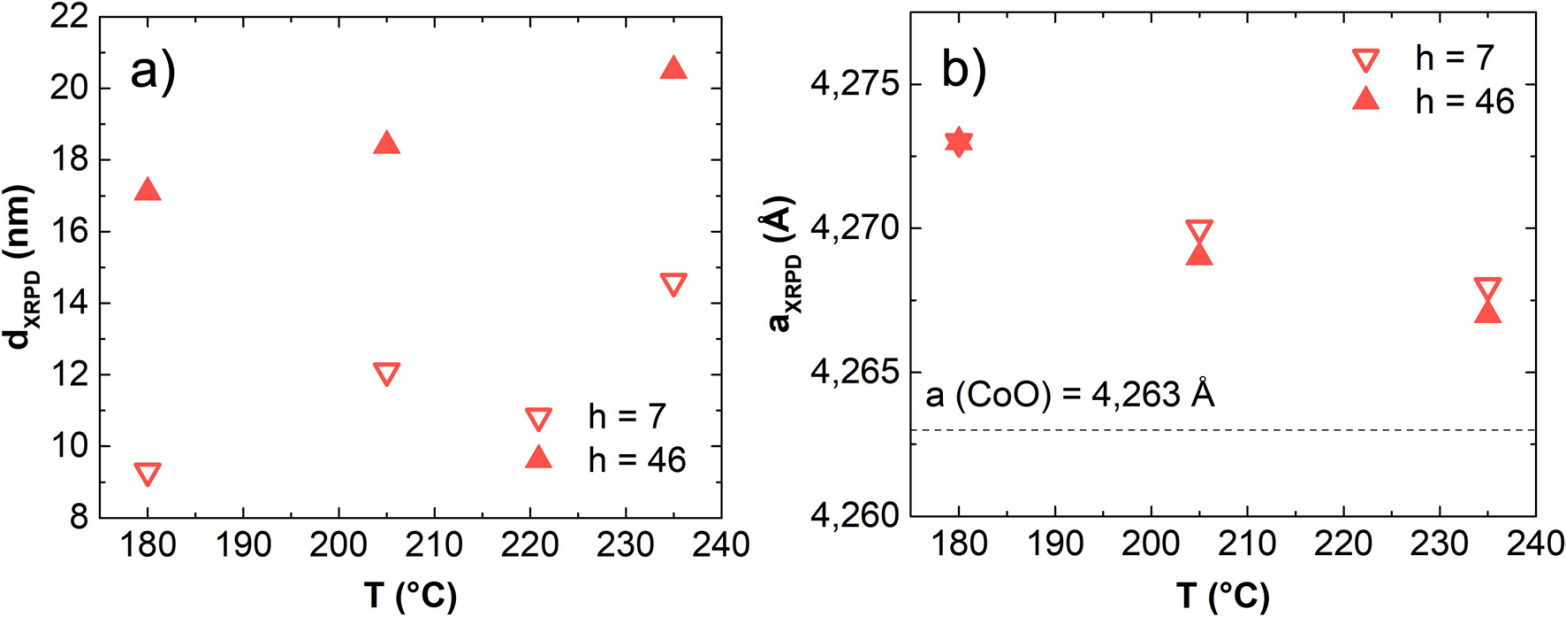
(**a**) XRPD crystallite diameters and (**b**) cell parameters for TTEG samples changing with T and h.

**Figure 4:**
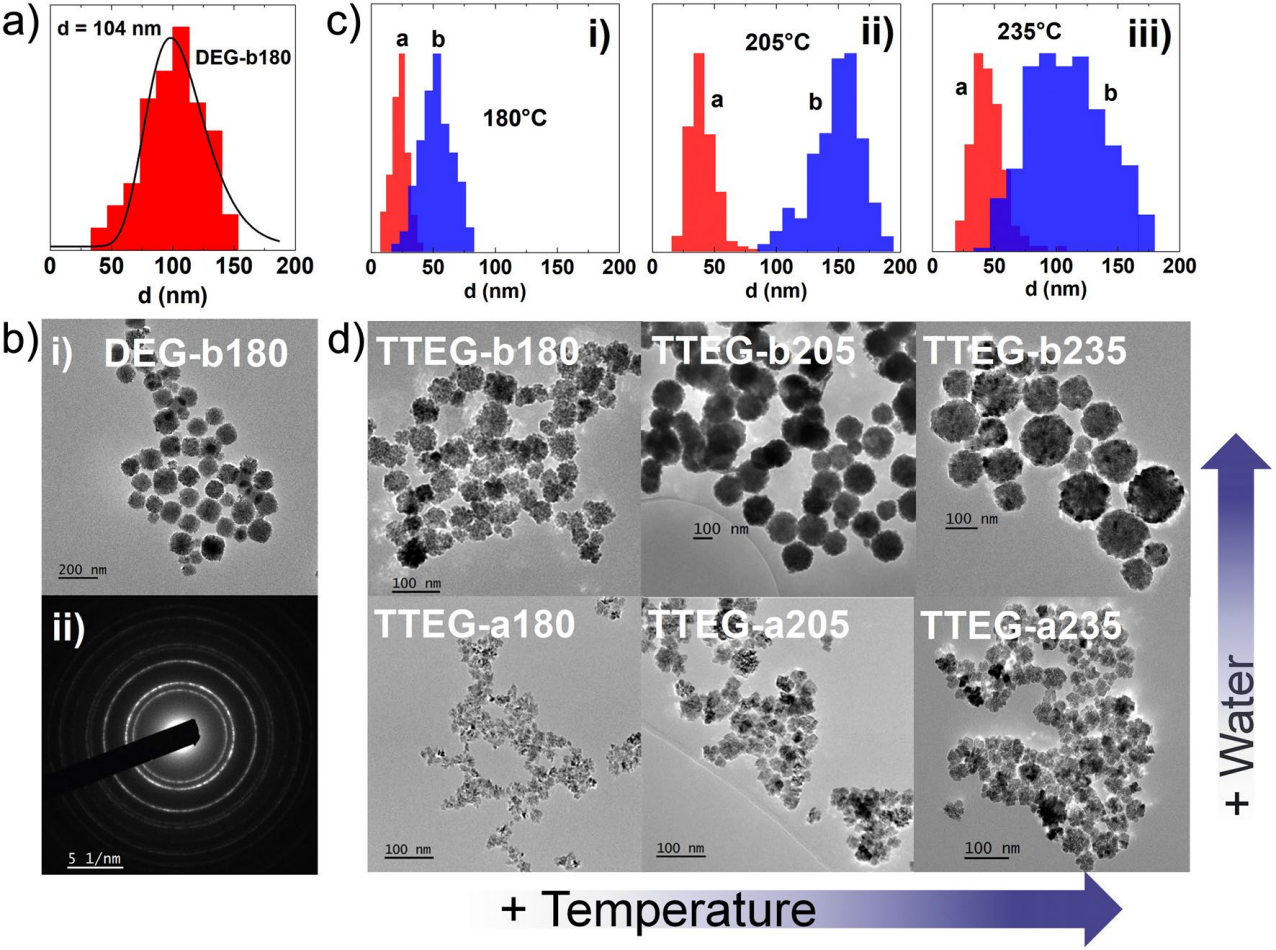
(**a**) size distribution of DEG1b, (**b**) (i) TEM image and (ii) SAED pattern of DEG1b, (**c**) size distribution of TTEG samples at two hydrolysis ratios compared for (i) 180 °C, (ii) 205 °C and (iii) 235 °C syntheses and (**d**) a panel of TEM pictures of each TTEG sample.

DEG syntheses demonstrated limited flexibility, whereas other polyols yielded more interesting results. Pure CoO was consistently obtained at higher temperatures with all the other polyols, and remarkable differences were produced at the two hydrolysis ratios used. Notably, the only non-DEG syntheses not producing pure CoO according to XRPD analysis are TEG-b180, TEG-b235 and PEG-b180. In TEG-b180, LHS residues were evident to XRPD analysis. The second case exhibited a combination of is *fcc*-CoO and *hcp* wurtzite-like CoO. Finally PEG-b180 likely contains brucite-like β-Co(OH)_2_, supported by the narrow IR adsorption at ~ 3500 cm^−1^ (Figure S8 and Refs.^61,62^). Among the syntheses yielding pure CoO, no interesting influences of the chosen synthesis parameters were found for TEG; conversely, in the case of PEG, octahedral aggregates and large crystalline size were obtained, especially at high h and temperatures (Figure S11). However, the most intriguing results emerged from experiments conducted with TTEG, the only solvent consistently yielding clean CoO in all cases (Fig. 2). Hence, we decided to dedicate additional attention on the samples produced in TTEG. Increases of temperature and hydrolysis ratios yielded an increase in the crystallite sizes, allowing to range between ~ 9 and ~ 20 nm (Fig. 3). In particular, at all the temperatures explored, a higher nominal hydrolysis ratio exerted a crystallite size increase of 6–8 nm. Both effects are also visible by TEM observation, where the aggregates sizes consistently increase with T and h (covering a range from ~ 25 to ~ 150 nm). Size effects are also observed in the primary crystal diameters (Fig. 4 and Table 1). Closer inspection of TEM results shows interesting morphology evolutions. Whereas in DEG only spherical aggregates of roughly spherical primary particles can be obtained (Fig. 4), longer polyols and higher temperatures exert morphological changes on both hierarchical levels. For example, in TTEG, spheroidal as well as octahedral primary particles can be produced, and gathered in to aggregates of remarkably different sizes. Further investigations on structure and morphology are conducted by means of HR-TEM microscopy.

**Table 1:** Hydrolysis ratio (h) and temperature (T) of the synthesis; cubic lattice parameters (a); average crystallite size (d_XRPD_); primary crystals size (d_TEM_); aggregates size (D_TEM_). Unities indicated in parentheses.

Sample	*h*	T (°C)	a (Å)	d_XRPD_ (nm)	d_TEM_ (nm)	D_TEM_ aggr. (nm)
DEG-b180	46	180	4.268	11	4	104
TTEG-a180	7	180	4.273	9	7	24
TTEG-a205	7	205	4.270	12	14	39
TTEG-a235	7	235	4.268	15	15	44
TTEG-b180	46	180	4.273	17	8	53
TTEG-b205	46	205	4.269	18	10	154
TTEG-b235	46	235	4.267	21	23	111
TTEG1	7	205	4.261	7	4	18
TTEG2	46	205	4.266	13	4	78

**Figure 5:**
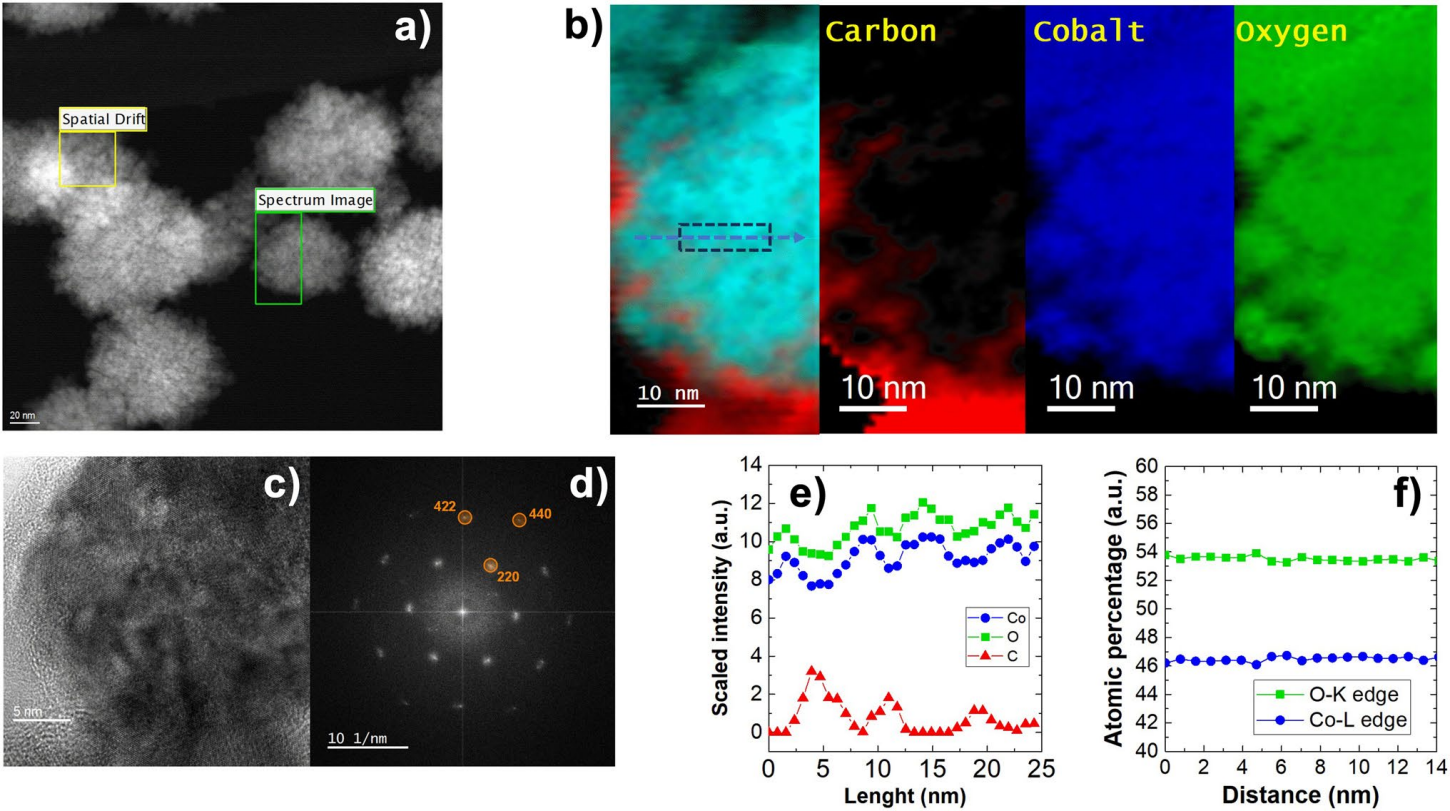
(**a**) STEM-HAADF pictures of the area of interest, (**b**) elemental maps of C, Co and O, (**c**) HR-TEM picture of a sample with its (**d**) FFT diffraction pattern, (**e**) EELS mapping and (**f**) relative and atomic percentages of Co and O.

EELS composition maps (Fig. 5) show the presence of carbon atoms on the aggregates’ surface, which are indicative of the presence polyol residues remained after the synthesis. Despite no impurity is detected in XRPD patterns, EELS provides evidence of a small excess of oxygen respect to a stoichiometric CoO. This is probably owed to a small quantity of spinel Co_3_O_4_ found in aged powders. However, the powders are checked again after the characterization, and no signs of the spinel phase are found (Figure S14), suggesting that its quantity is very small. SAED images of fresh samples (Fig. 4b-ii) confirm the absence of phases with spinel structure. Furthermore, FFT on fresh samples TEM images (Fig. 5) show only the spots of CoO, meaning that the oxidation occurred after the magnetic characterization, as discussed in the section about magnetism.

To have further clues about the reaction, more samples were synthesized by stopping the synthesis in TTEG at 120 °C, 165 °C and 180 °C. The synthesis is known to form an LHS intermediate at first^16^, after which such LHS dissolves to allow the nucleation of CoO to occur; despite this, it was possible to obtain a precipitate just in the h = 46 experiments. This could be a sign of a mechanism change due to the effect of water, which is relevant to the formation of the LHS intermediate^16^ and could explain the remarkable size differences in TTEG. Alternatively, water could be influencing the solubility of the precipitated phases, and act on CoO morphology mainly interacting with the mechanism proposed by Gaudisson et al.^21^. From XRPD it is observed that the sample evolution consists of two different phases. The phase present at 180 °C is a brucite like layered hydroxy-acetate salt with turbostratic disorder^61^, consisting of equally spaced, randomly oriented and positively charged Co(OH)_2-x_ layers separated by intercalary water and acetate molecules. XRPD pattern and the TEM picture (similar to the turbostratic structures observed by Poul et al. in Ni-LHS^25^) confirm this explanation. The reflections from ~ 38° are attributed to the (hk0) planes diffractions generated by the Co(OH)_2-x_ layers, while anything before that can be attributed to the 00 l reflections (i.e., the space between the layers^61^). FTIR studies on cobalt acetate^63,64^ allowed us to identify the phase precipitated at 120 °C as less hydrated cobalt acetate (e.g., Co(ac)_2_·0.5H_2_O).

### Magnetic study

**Figure 6:**
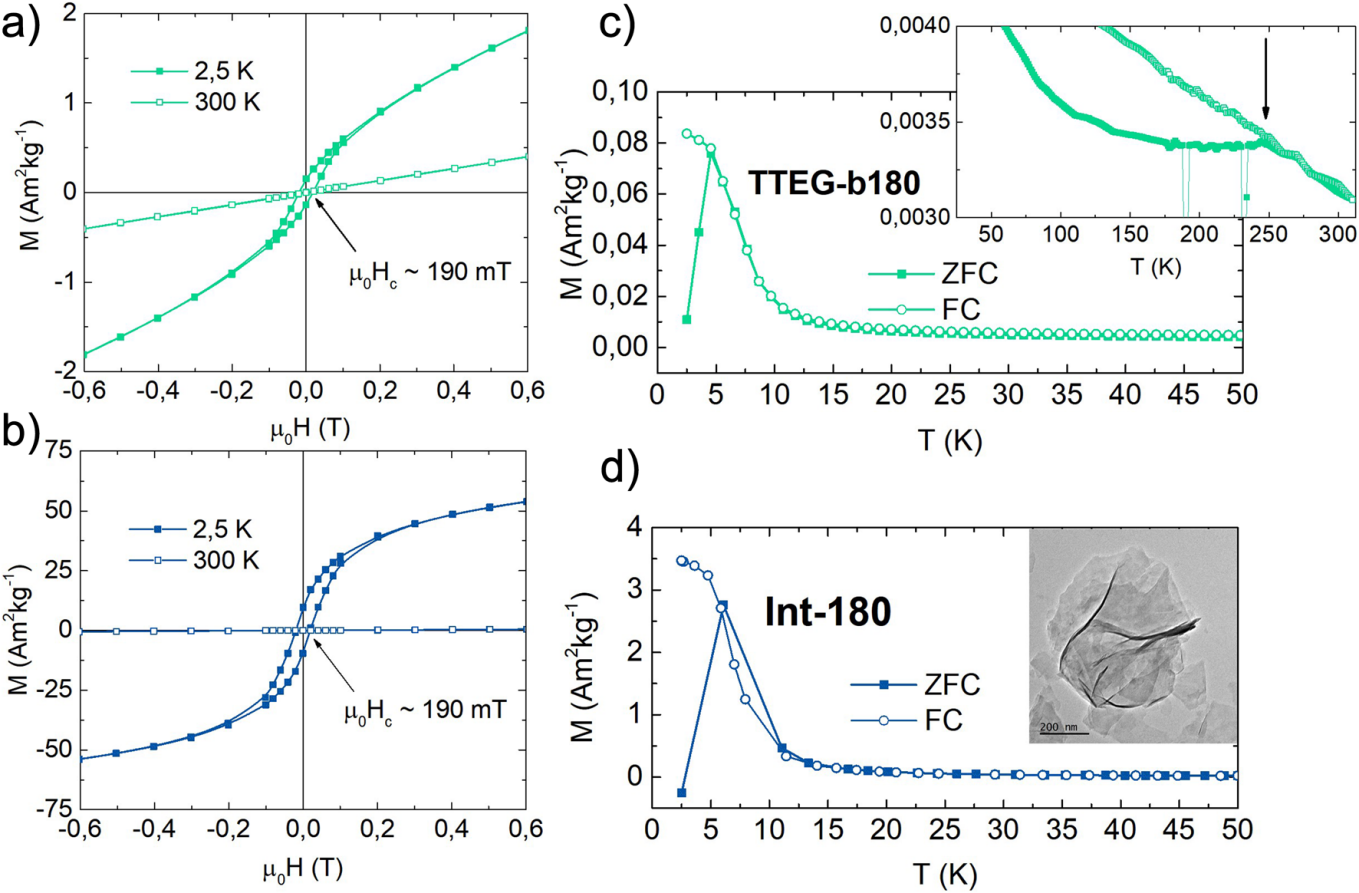
Isothermal magnetization plots at 2.5 K and 300 K of (**a**) TTEG-b180 and (**b**) Int-180, and ZFC/FC curves of (**c**) TTEG-b180 (50–300 K zoom in the inset) and (**d**) Int-180 (Int-180 TEM picture in the inset).

Field and temperature dependence of magnetization have been investigated for all the samples. TTEG-b180 was studied by means of isothermal magnetization plots (MVsH) at 2.5 and 300 K, and ZFC/FC curves (Fig. 6). Irrespective of CoO being AFM, its 2.5 K MVsH plots showed a non-saturating character at high field with a ~ 190 mT coercivity (μ_0_Hc), while ZFC/FC curves with an extremely low T_max_ (~ 7 K), and no signs of CoO’s T_N_. Despite the absence of any visible impurity on the XRPD pattern, MVsH and MVsT measurements suggest the superimposition of a FM and an AFM behavior. This landscape matches quite well the result obtained on Ni based LHS materials by Taibi et al.^53^, suggesting presence of LHS ferromagnetic residues in quantities too low to be detected by XRPD. This hypothesis was tested by stopping the synthesis at its beginning at ~ 180 °C to investigate the products’ magnetic properties (sample Int180, Fig. 6d). Int-180 sample exhibits the same coercivity and a T_max_ values as those observed in TTEG-b180, confirming that the magnetic properties of AFM CoO are, indeed, dominated by the ferromagnetic LHS. It is wort to underline that, as expected, almost no contribution is observed at room temperature where a typical antiferromagnetic behavior is shown (Fig. 6).

**Figure 7:**
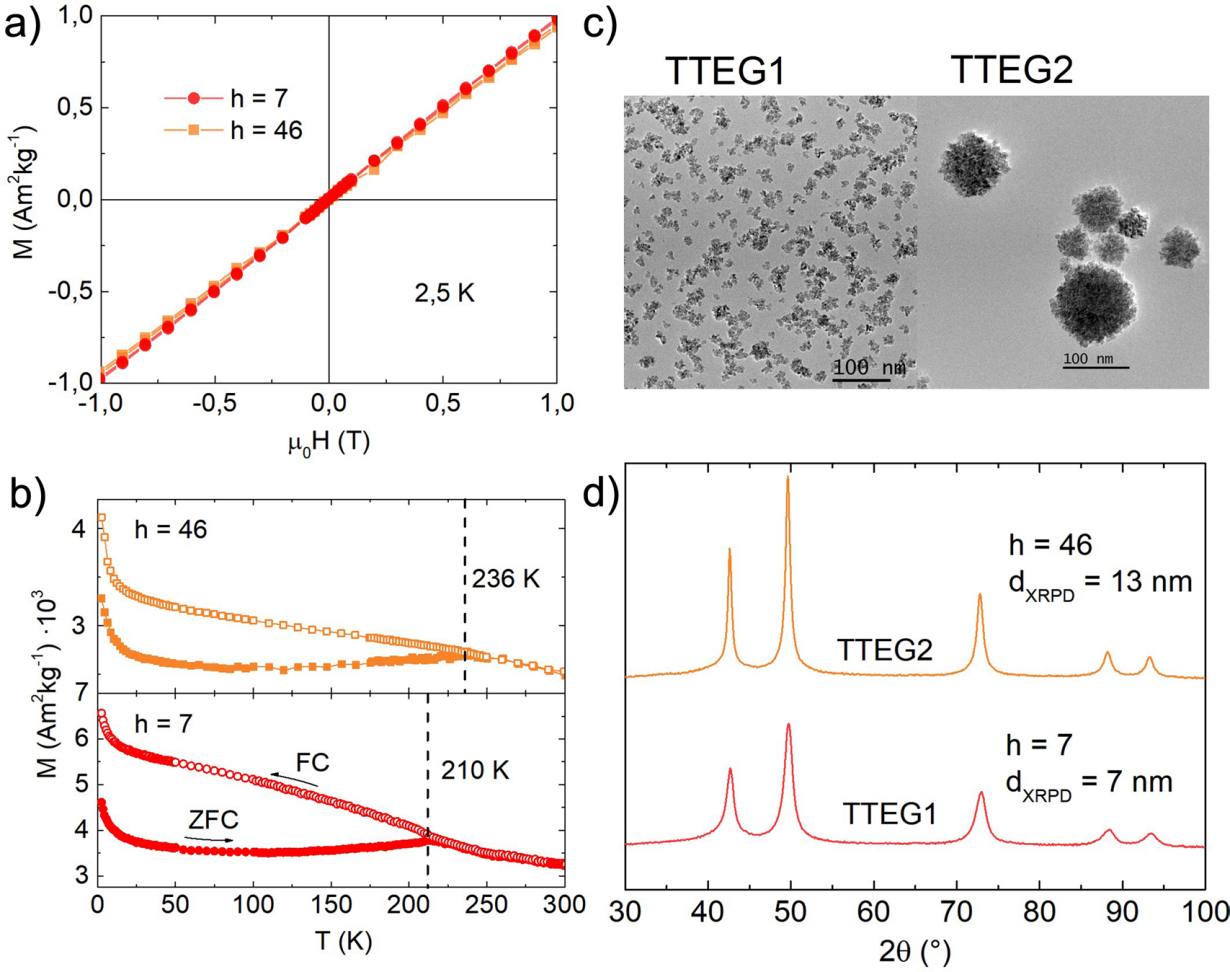
(**a**) 2.5 K MVsH plots, (**b**) ZFC/FC curves, (**c**) TEM pictures and (**d**) XRPD patterns of TTEG1 and TTEG2.

**Figure 8:**
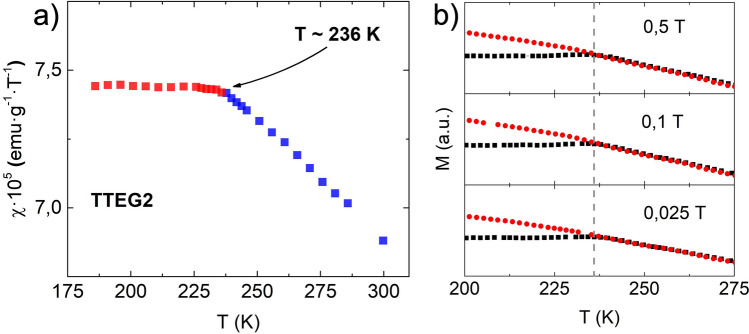
(**a**) Susceptivity and (**b**) multiple fields ZFC/FC of TTEG2.

Several papers on CoO produced by different synthesis techniques report a ZFC maximum in temperature range 5–10 K^65–69^. In the studies, the peak is given with various interpretations, such as the blocking of CoO nanocrystals^65,67^, small Co_3_O_4_ NPs^66^, shape induced ferromagnetic phenomena^68^, and non-compensation of surface spins^69^. Since layered structures can form quite easily with many chemicals—such as long organic surfactants or small inorganic molecules^53,61,70–72^—we believe that this and other magnetic features observed on the cited literature can be ascribed to the formation of layered magnetic structures, such as LHS or layered double hydroxides (LDH)^61^. Upon closer inspection, nothing meaningful was found at ~ 290 K, but another T_max_ was also found in TTEG-b180 at ~ 250 K (Inset Fig. 6c). The meaning of this feature will be explained later. LHS-free CoO was obtained by reducing to one half the concentration of the precursor, producing two other samples, namely TTEG1 and TTEG2, respectively at h = 7 and 46, exploiting the effect of water to obtain samples of different sizes. In these samples, the expected antiferromagnetic behavior has been observed at 2,5 (Fig. 7). As previously discussed, a small excess of oxygen, likely due to surface ageing oxidation, is found in with EELS analysis. Co_3_O_4_ is known to induce hysteretic behavior at low temperature in CoO^73–75^. However, samples investigated with HR-TEM are checked after the analysis with XRPD, where no signs of its presence are found; furthermore, no signs of the magnetic transition temperature of Co_3_O_4_ (T_N_ ~ 40 K^76^) can be found on any magnetic measurements, meaning that the spinel was formed in the time between the magnetic measurements and EELS, and its quantity is very small. As a final proof, we point out that SAED pattern measured on fresh samples (Fig. 4) shows only the diffraction rings of fcc-CoO. Thus, we can interpretate all our magnetic results neglecting any spinel presence. ZFC/FC curves show a steep increase of the magnetization at very low temperatures—owed to paramagnetic contributions coming from uncompensated surface magnetic moments^47^—and a T_max_ where the two curves meet, which increases with the hydrolysis ratio and—hence—with the nanoparticles’ size. To give a meaning to the temperature several characterizations, susceptibility and ZFC/FC at different fields were measured on TTEG2. All the characterizations clearly evidence a transition at T_max_. The change in slope in the susceptibility measurements is indicative of an antiferromagnetic to paramagnetic transition, while the T_max_ from the multiple field ZFC (Fig. 8) shows no dependence on the field magnitude, meaning that the transition cannot be ascribed to a magnetic dynamic magnetic phenomenon (e.g., superparamagnetic relaxation or spin glass^77,78^). For these reasons, the temperature was interpreted as the T_N_ of the sample, which would be thus tunable by simple action on the water content, in the case of TTEG, and by acting on simple synthesis parameters in other cases.

#### Size dependence of Néel temperature

**Figure 9:**
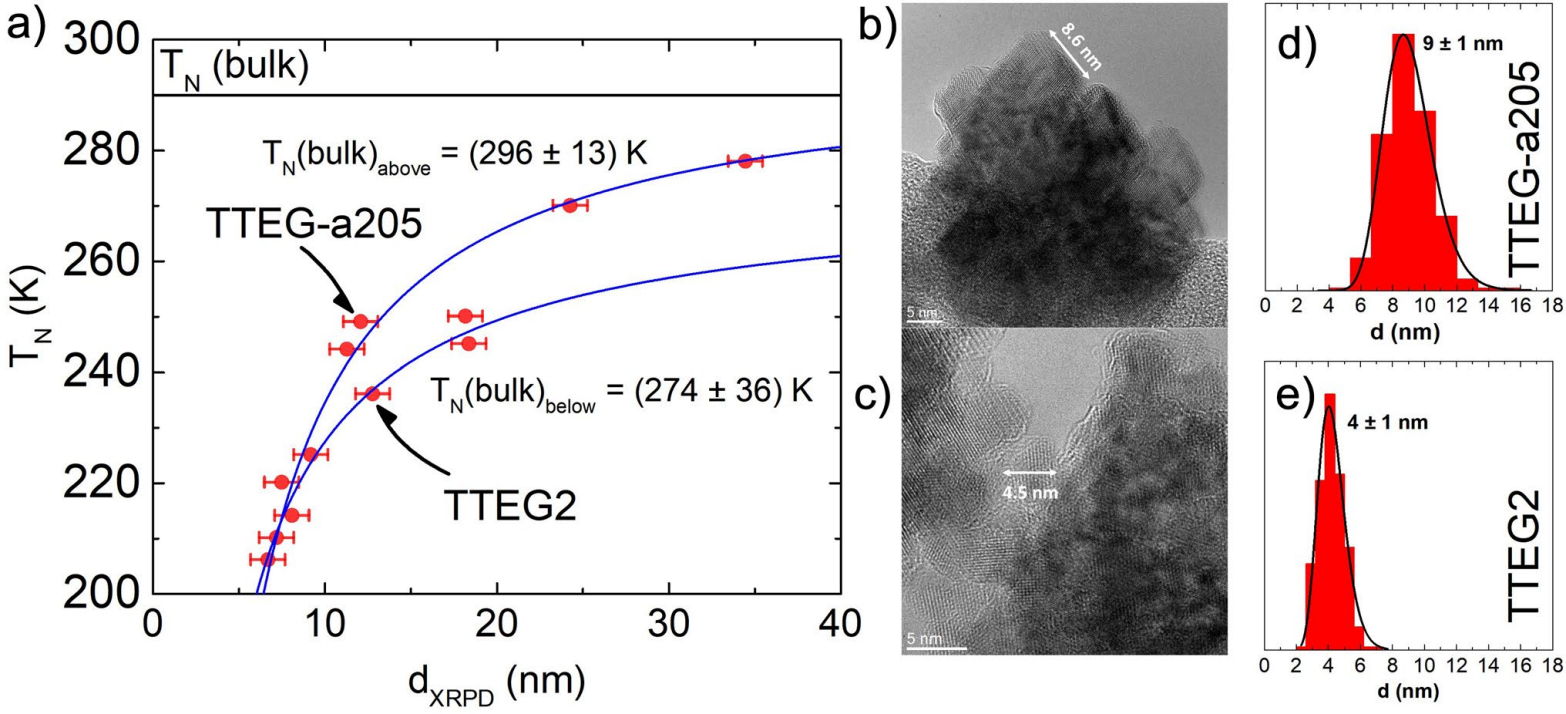
(**a**) Néel Temperature Vs crystalline length curve, HR-TEM pictures of (**b**) TTEGa2 and (**c**) TTEG2, and primary crystal size distribution of (**d**) TTEG-a205 and (**e**) TTEG2.

To further investigate the dependence of T_N_ from crystallite size, we selected several samples covering the whole range of crystallite sizes that we have produced in our experiments. ZFC/FC curves were measured on these to know their T_N_, and all the results were plot with respect to the XRPD crystallite size. The decrease in the magnetic critical temperature (T_N_ or T_C_) with size reduction has been already observed, especially on thin films of various materials – such as NiO, CuO, Ni, Ho, Fe_3_GeTe_2_ and others^79–85^. In general, ordered magnetic phenomena are caused by exchange/super-exchange interactions between electrons, forcing the atomic magnetic moments to align in certain relative directions; however, the interaction acts within a correlation length larger than the distance between two first neighboring cations. Thus, when the material size decreases, several super-exchange interactions are lost (especially near the surface), leading to a general weakening of the magnetic order and a decrease in the critical temperature^86–88^. In the case of thin films, the phenomenon was mathematically formulated by Zhang and Willis^81^, who proposed that the atoms of the film lose a certain number of primary and subsequent neighboring interactions, dependent on the distance from the surface. At smaller scales, the reduced number of atoms becomes significant, affecting the fundamental properties of the material:

$${T}_{N}\left(d\right)={T}_{N}\left(bulk\right)\cdot \left[1-{\left(\frac{\xi }{d}\right)}^{\lambda }\right]$$

where ξ is the spin–spin correlation length, and λ is a shift exponent. In our case, basing on XRPD diameters ranging from ~ 8 to ~ 35 nm, we observed T_N_ ranging from ~ 205 to ~ 280 K (Fig. 9). Interestingly, the curve splits into two branches, with approximately 15 K of difference in the 12–20 nm range. By fitting only the upper branch with equation, a bulk T_N_ of 296 ± 13 K is obtained, close to the literature value of 290 K; the lower branch provides a bulk value of 273 ± 36 K).

The curve splitting is obtained also when the size is estimated with other methods, such as Scherrer equation or Williamson-Hall plot function of High Score Software, with the same samples falling on the lower branch of the graph. In our opinion, this effect could be a result of the presence of defects forming during the synthesis and breaking the spin–spin correlation paths to a further extent. This would be consistent with the lower ξ value (2.0 nm for the upper curve, 1.4 nm for the lower one), and with previous studies, showing that that the concentration of vacancies can reduce T_N_^89^. To clarify this aspect, two samples (namely TTEG2 and TTEG-a205) are compared. Geometric Phase Analysis (GPA, Figure S13) does not show substantial differences in terms of strain, and does not disambiguate the phenomenon. However, TTEG2 and TTEG-a205 still have visible morphological differences: while TTEG2 has spheroidal primary crystals of ~ 4 nm, TTEG-a205 is composed of faceted primary crystals of ~ 8 nm. We infer that the smaller primary crystal size of TTEG2 crystals might be a sign of less order within the aggregate, and that the two primary crystal shapes might result in a difference in terms of packing factor, which is higher for faceted crystals; this could be causing the presence of voids inside TTEG2, cutting the super-exchange correlation paths in the aggregate. In fact, all the particles belonging to the lower branch have a spheroidal primary shape, none of them being faceted (Figure S12). If this effect is confirmed, it could be a new and interesting way to tune the materials’ magnetic properties, likely applicable also to ferromagnetic materials.

## Conclusions

In conclusion, we have studied the polyol synthesis of fcc-CoO nanoparticles by investigating the effects of polyol length and temperature at two different hydrolysis ratios, thus, obtaining interesting structural and morphological influences, i.e., crystal sizes ranging from ~ 8 to ~ 40 nm, aggregate sizes from ~ 20 to ~ 150 nm, and new morphologies, such as nanoflowers and octahedra. Also, the 180 °C reaction limit imposed by the solvent was extended to 235 °C and possibly beyond, up to the solvent boiling temperature. New mechanistic clues about the polyol synthesis are provided by our studies. All these information make the polyol synthesis of CoO tunable with a lot more freedom and are possibly extendable to the synthesis of other transition metal oxides. From the perspective of magnetism, the effects of Co-LHSs was separated from the magnetic properties of pure CoO, also providing us with interpretations of other results obtained in literature. The maximum temperature in ZFC characterizations was interpreted as the Néel temperature of the materials, allowing us to observe its decrease for smaller particle sizes. A regular dependence of T_N_ on the crystal coherence diameter is observed, and clues for further degrees of freedom acting on T_N_ are found. As a final remark, water has been shown to be an effective tool to tune the synthesis result on a morpho-structural level and provided us with a tool to act on the synthesis very simply in order to modify the magnetic properties of the result.

### Supporting information

Additional experimental details, materials, and methods. It includes synthesis quantities and conditions, a panel of XRPD patterns of all the syntheses tried in each experimental condition, TGA curves, HR-TEM and STEM-HAADF pictures, EELS and GPA analyses.

### Supplementary Information

Supplementary Information.

## Data Availability

The dataset used and/or analyzed during the current study is available from the corresponding author on reasonable request.
