# Supplementary material for: Advancements in polyol synthesis: expanding chemical horizons and Néel temperature tuning of CoO nanoparticles
Source: Sci Rep. 2024 May 31;14:12529. doi: 10.1038/s41598-024-54892-2 (PMC11143313; doi:10.1038/s41598-024-54892-2)
Supplement: Supplementary file 1 — Supplementary Information. [file 41598_2024_54892_MOESM1_ESM.docx]

Supplementary material – Advancements in Polyol Synthesis: Expanding Chemical Horizons and Néel Temperature Tuning of CoO

# Polyol synthesis

The syntheses are performed in a 250 mL three necked flask. The quantities for a standard synthesis are showed in **Table S1**, with the hydrolysis ratio. All the chemicals were purchased from Sigma Aldrich. Co(ac)_2_·4H_2_O has a ≥98.0% purity, while DEG, TEG and TTEG have a 99% purity; PEG has an average molecular mass of 400 gmol^-1^, further purity indications are not available; distilled water was employe. The synthesis is typically performed in a 250 mL three necked flask, with 100 mL of polyol and a mechanical stirrer set to 450 rpm.

Table S 1: chemicals’ quantities, hydrolysis ratio, synthesis time and heating rate for a standard synthesis

| **Co(ac)_2_·4H_2_O** | 0.1 mol/L | |
| --- | --- | --- |
| **nEG** | 100 mL | |
| **H_2_O** | 2.80·10^-4^ mol/L | 4.16·10^-3^ mol/L |
| ***h*** | 7 | 46 |
| **time** | 18 h | |
| **heating rate** | 6° C/min | |

The experiments done include the exploration of three variables, such as polyol length, temperature, and hydrolysis ratio *h*. A scheme of the experimental space explored can be found in **Figure S1**.


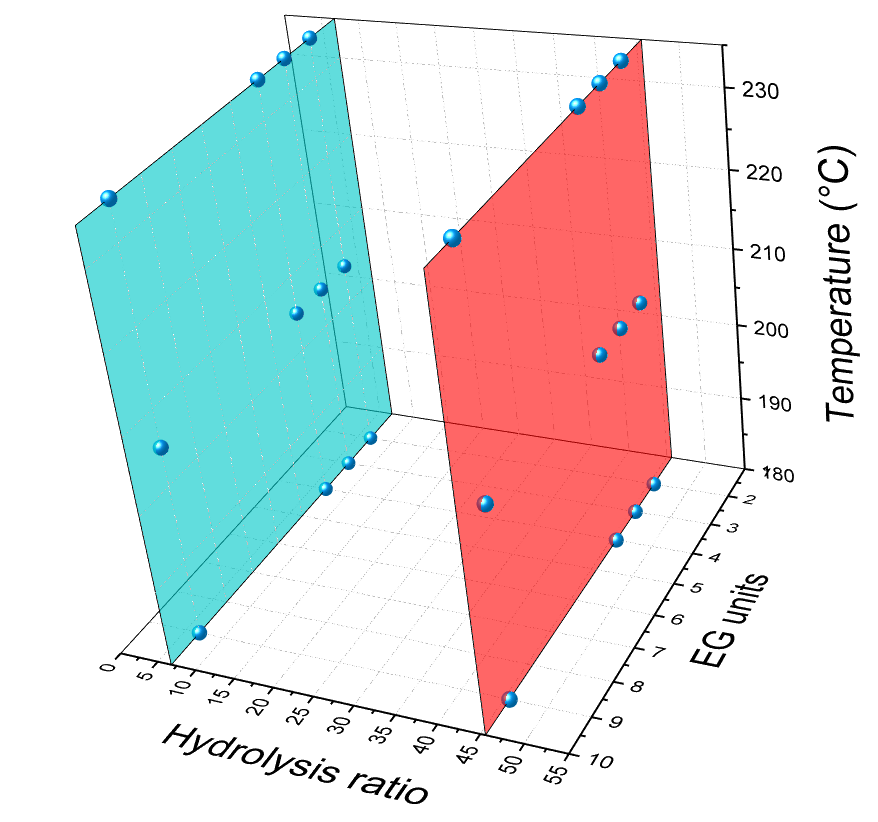


Figure S 1: a scheme of the experiments and the variables explored.

To obtain TTEG1 and TTEG2 samples, the precursor’s concentration was halved, and the hydrolysis ration kept the same, according to **Table S2**. Other experiments have shown that 18 h, as previously proposed in literature^1^, are not strictly necessary to achieve CoO formation. This is the reason why the synthesis was shortened to 3 h. We cannot assume that with other polyols such a short time will be enough to eliminate LDH intermediates, but due to its small quantity, for purposes other than studying magnetic properties, it is not a problem.

Table S 2: chemicals’ quantities, hydrolysis ratio, synthesis time and heating rate for TTEG1 and TTEG2

| **Co(ac)_2_·4H_2_O** | 0.05 mol/L | |
| --- | --- | --- |
| **nEG** | 100 mL | |
| **H_2_O** | 1.40·10^-4^ mol/L | 2.08·10^-3^ mol/L |
| ***h*** | 6.8 | 45.6 |
| **time** | 3 h | |
| **Temperature** | 205° C | |
| **heating rate** | 6° C/min | |

# Structure (XRPD) and morphology (TEM)

Table S 3: all the syntheses executed, their experimental conditions, and morphostructural results

| **Sample** | **Polyol** | ***h*** | **T**  **(° C)** | **a (Å)** | **d_XRPD_ (nm)** | **D_TEM_ aggr. (nm)** | **Outcome** |
| --- | --- | --- | --- | --- | --- | --- | --- |
| **DEG-a180** | DEG | 7 | 180 | - | - | - | Amorp. C |
| **DEG-a205** |  | 7 | 205 | - | - | - | Amorp. C+CoO |
| **DEG-a235** |  | 7 | 235 | - | - | - | Co_n_C |
| **DEG-b180** |  | 46 | 180 | 4.268 | 11 | 102 | CoO |
| **DEG-b205** |  | 46 | 205 | - | - | 53 | CoO+Co_n_C |
| **DEG-b235** |  | 46 | 235 | - | - | - | Co_n_C |
| **TEG-a180** | TEG | 7 | 180 | 4.271 | 10 | 113 | CoO |
| **TEG-a205** |  | 7 | 205 | 4.273 | 9 | 73 | CoO |
| **TEG-a235** |  | 7 | 235 | 4.270 | 9 | 88 | CoO |
| **TEG-b180** |  | 46 | 180 | - | - | - | LDH+CoO |
| **TEG-b205** |  | 46 | 205 | 4.265 | 7 | 17 | CoO |
| **TEG-b235** |  | 46 | 235 | - | - | 119 | CoO+hexCoO |
| **TTEG-a180** | TTEG | 7 | 180 | 4.273 | 9 | 24 | CoO |
| **TTEG-a205** |  | 7 | 205 | 4.270 | 12 | 39 | CoO |
| **TTEG-a235** |  | 7 | 235 | 4.268 | 15 | 44 | CoO |
| **TTEG-b180** |  | 46 | 180 | 4.273 | 17 | 53 | CoO |
| **TTEG-b205** |  | 46 | 205 | 4.269 | 18 | 154 | CoO |
| **TTEG-b235** |  | 46 | 235 | 4.267 | 21 | 111 | CoO |
| **PEG-a180** | PEG | 7 | 180 | 4.268 | 23 | 48 | CoO |
| **PEG-a205** |  | 7 | 205 | 4.267 | 24 | 50 | CoO |
| **PEG-a235** |  | 7 | 235 | 4.267 | 26 | 56 | CoO |
| **PEG-b180** |  | 46 | 180 | - | - | - | unidentified |
| **PEG-b205** |  | 46 | 205 | 4.271 | 18 | 46 | CoO |
| **PEG-b235** |  | 46 | 235 | 4.267 | 35 | 66 | CoO |


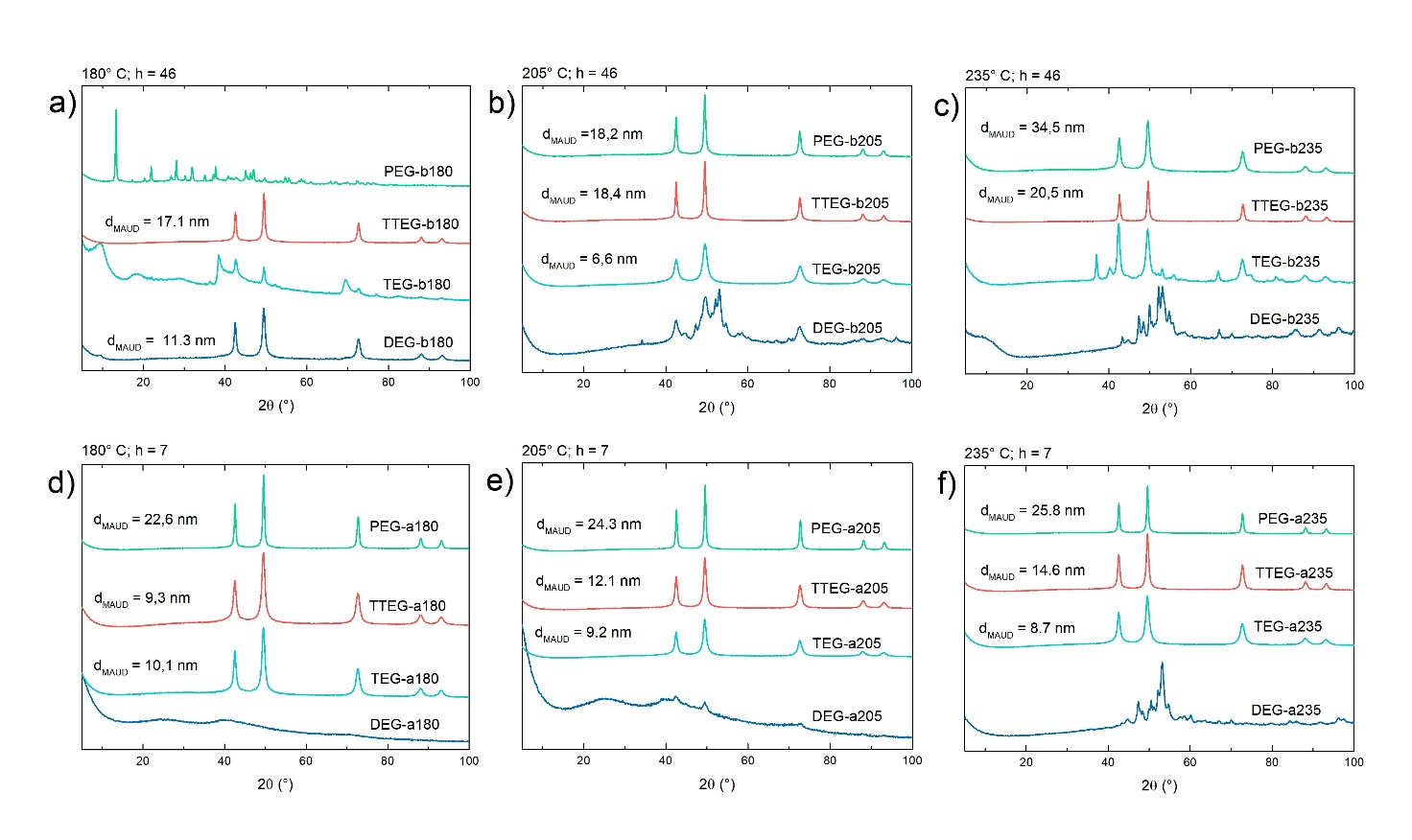


Figure S 2: XRPD patterns all the synthesis tried with different polyols (blue for DEG, turquoise for TEG, red for TTEG, green for PEG) at a) 180° C and h = 46 b) 205° C and h = 46 c) 235° C and h = 46 d) 180° C and h = 7 e) 205° C and h = 7 f) 235° C and h = 7.

# TGA

Thermogravimetric Analysis (TGA) was performed on few selected samples. In the case of TTEG1 and TTEG2, the difference in mass loss can be related to the different aggregates size, which in the case of TTEG1 is smaller, thus leaving more surface to be covered by the polyol. The heat flow shows two superimposed peaks, which can be attributed to the polyol loss and to the sample oxidation from CoO to Co_3_O_4_.


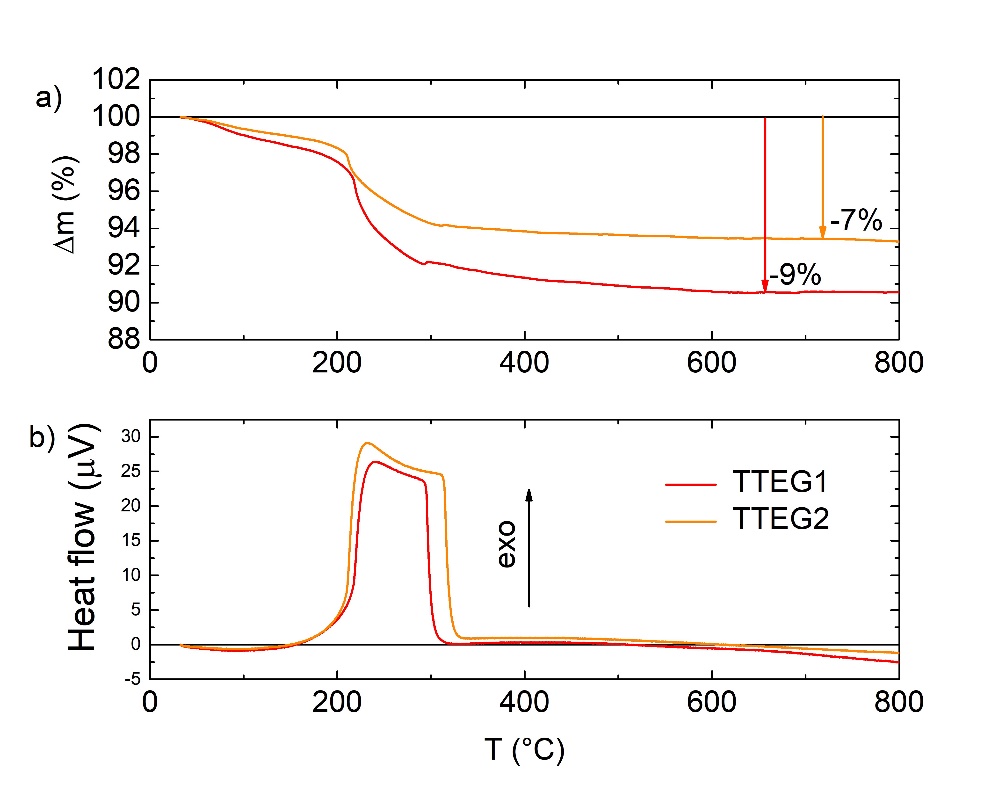


Figure S 3: a) mass loss and b) heat flow recorded during TGA.

The analysis is also performed on the synthesis intermediates, which show a more visible mass loss respect to the CoO sample. This is because the intermediate structures contain relatively volatile and decomposable molecules – such as water or acetates. Interestingly, Int02 shows a greater mass loss compared with Int06. Both TTEG2 and Int06 bring to the formation of Co_3_O_4_ as final product.


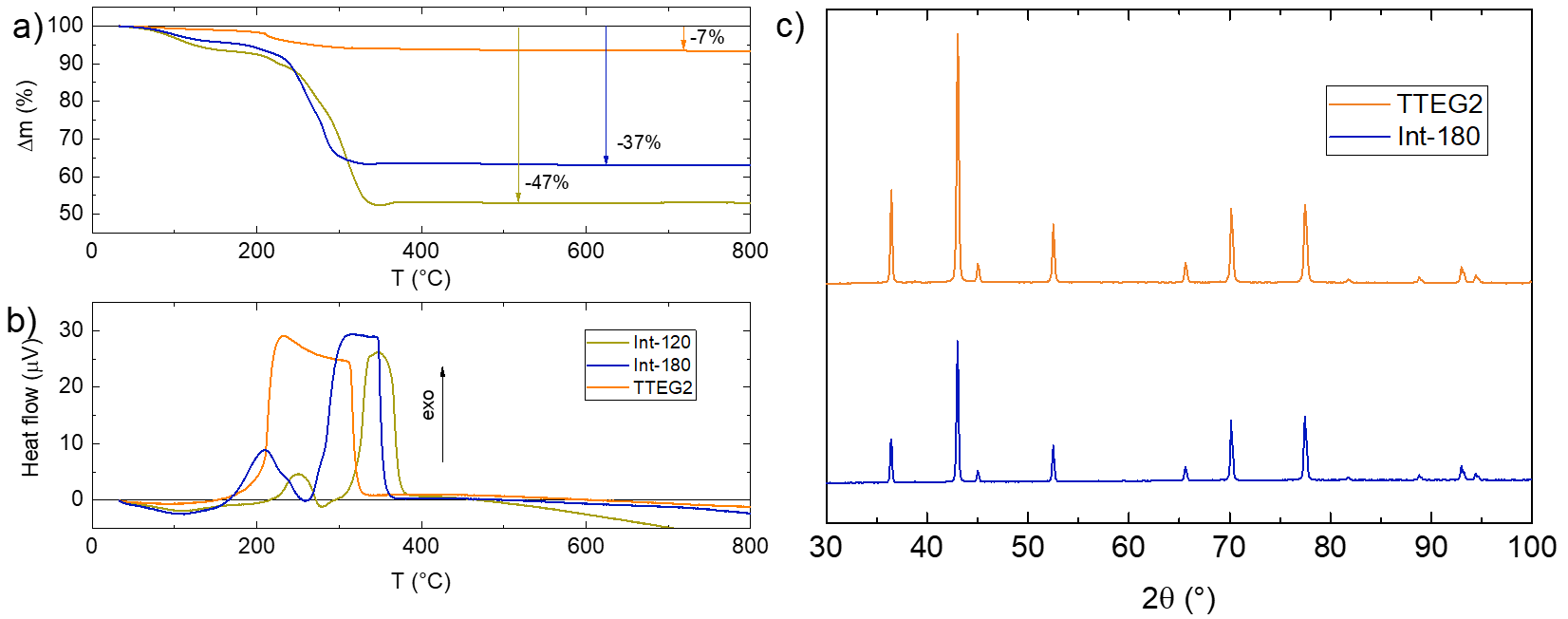


Figure S 4: TGA of intermediates compared with the one of TTEG2.

# Intermediates

LHS phases are normally formed before the nucleation of CoO, an also probably play a fundamental role in the synthesis mechanism.


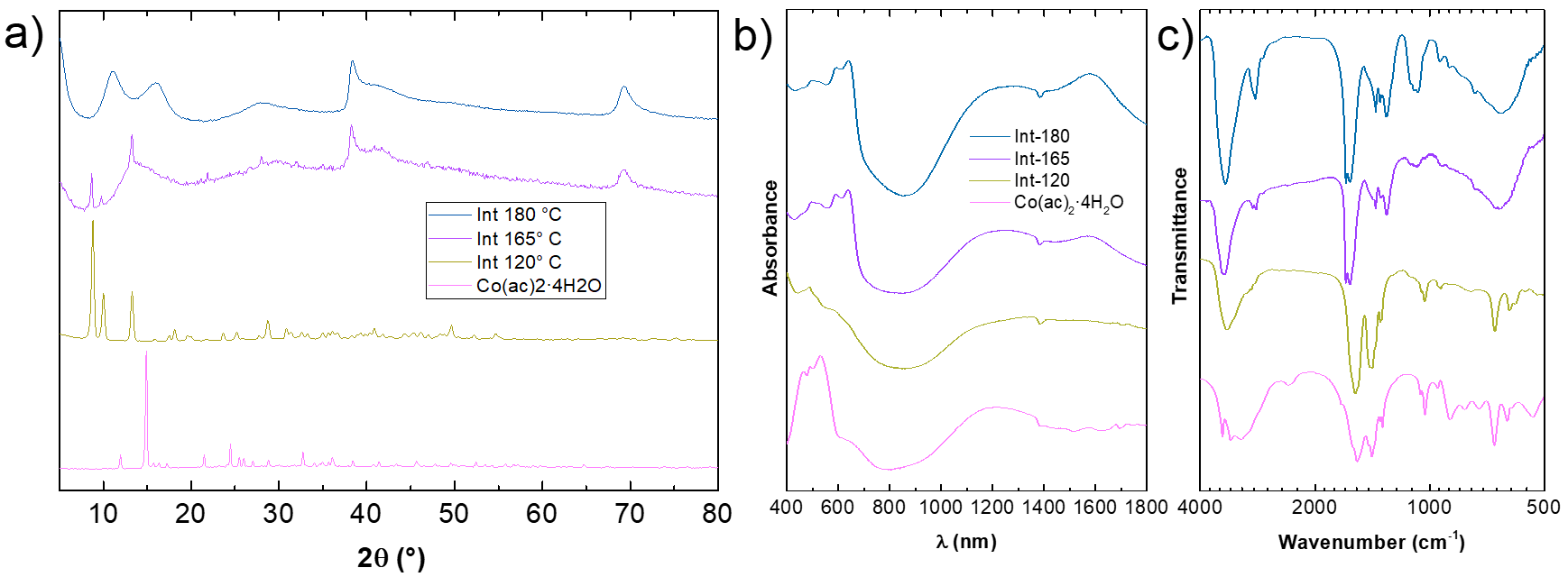


Figure S 5: a) XRPD, b) UV-Vis and NIR and c) IR spectra of the intermediate evolution at the early stages of the synthesis.

As much as for the TTEG syntheses, also the other polyol intermediates were prepared by stopping the synthesis protocol at 180° C. Apart from PEG, all of them showed the typical XRPD pattern of an LHS structure with turbostratic disorder. From ~ 40°, where the (hk0) peaks belonging to the cobalt hydroxide layer start to appear, no remarkable differences are spot between the samples. Instead, lower angle peaks usually attributed to (00l) peaks show some changes, suggesting that the use of the polyol probably changes the interlayer space, with possible influences on the LHS stability.


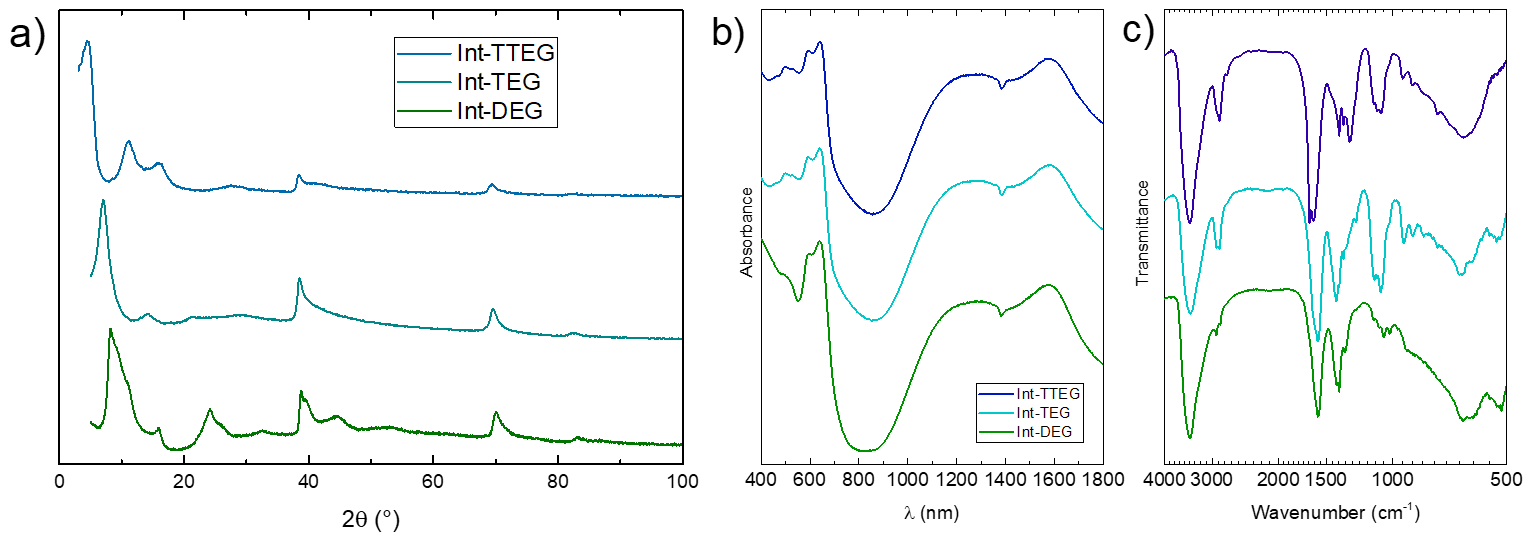


Figure S 6: a) XRPD patterns b) UV-Vis and c) FTIR spectra of Co-LHS intermediates in different polyols. Int-TTEG is called Int-180 in the main text.

The same experiment is done with PEG, but the LHS phase was not formed; instead, the XRPD pattern shows the formation of the same phase as in what happens for Int120, which was interpretated as less hydrated acetate. This suggests that the secondary acetate formation might be a common passage in the synthesis, at least for longer polyols, as well as a different precursor solubility.


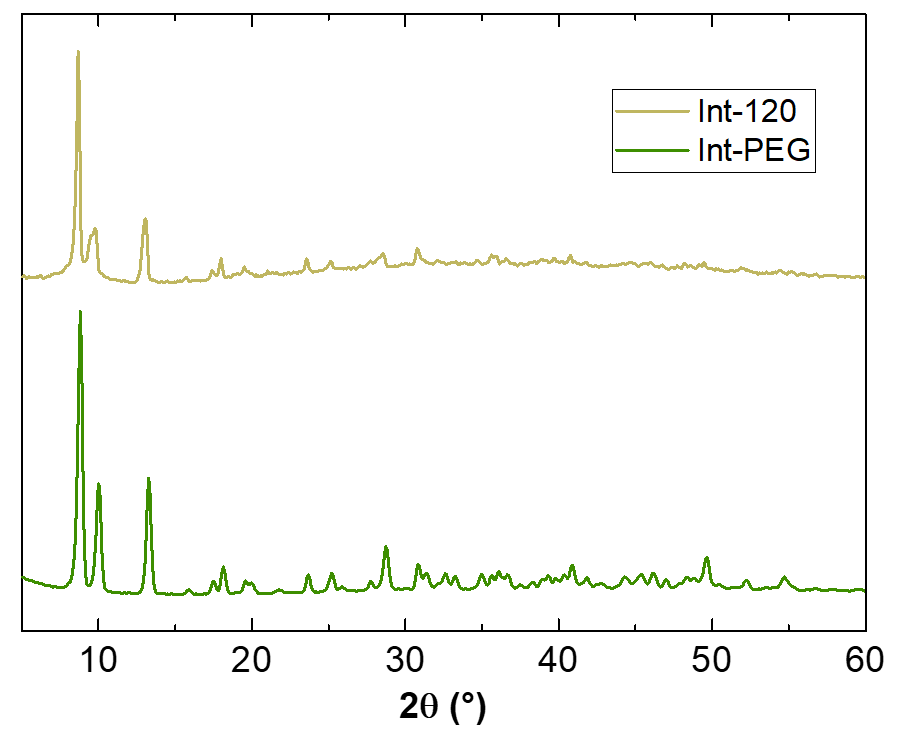


Figure S 7: XRPD patterns of Int-120 and Int-PEG.


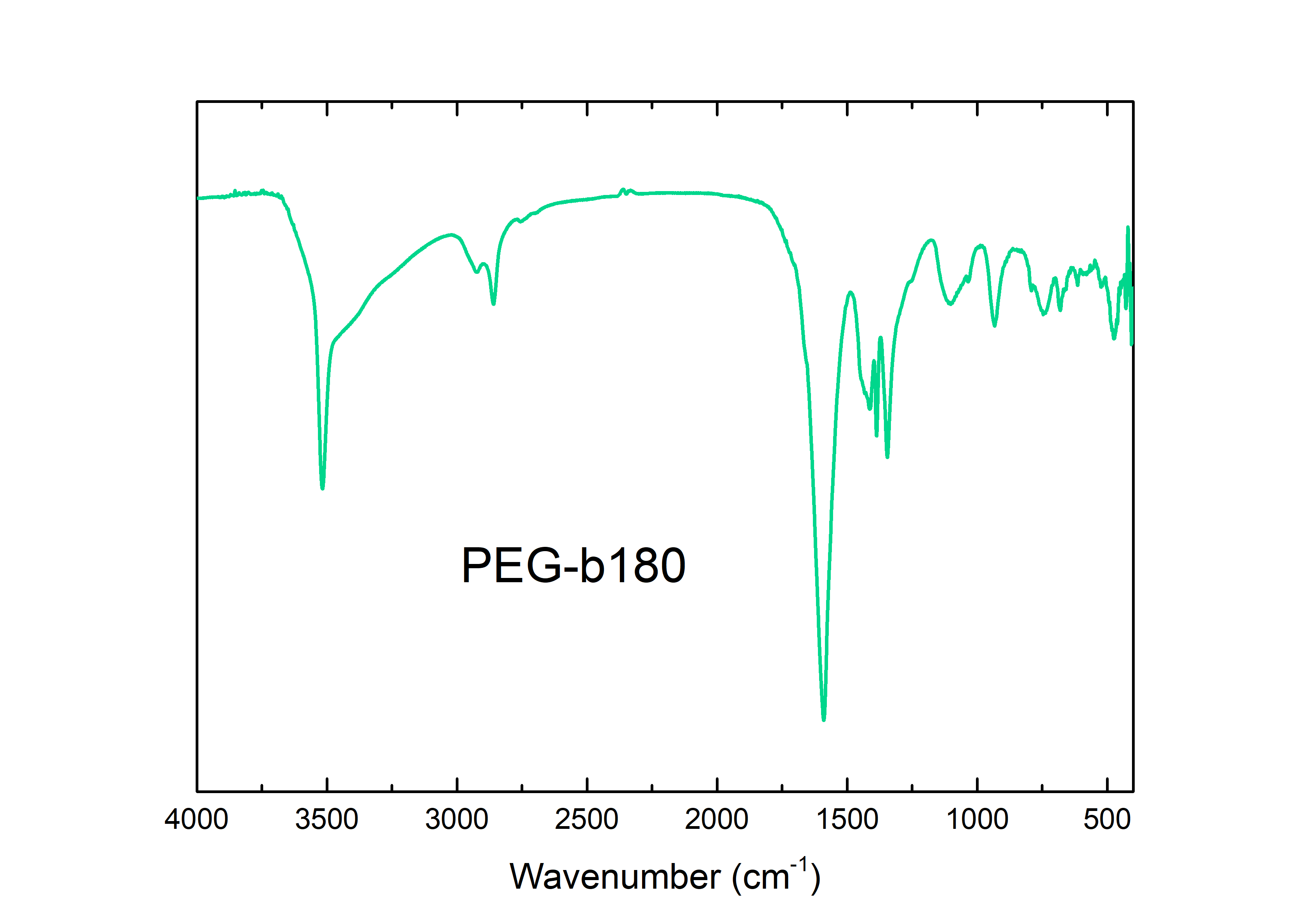


Figure S 8: FTIR of PEG-b180

# TEM, HR-TEM and STEM-HAADF pictures

HR-TEM pictures allow to observe the crystal alignment noted in literature^2^.


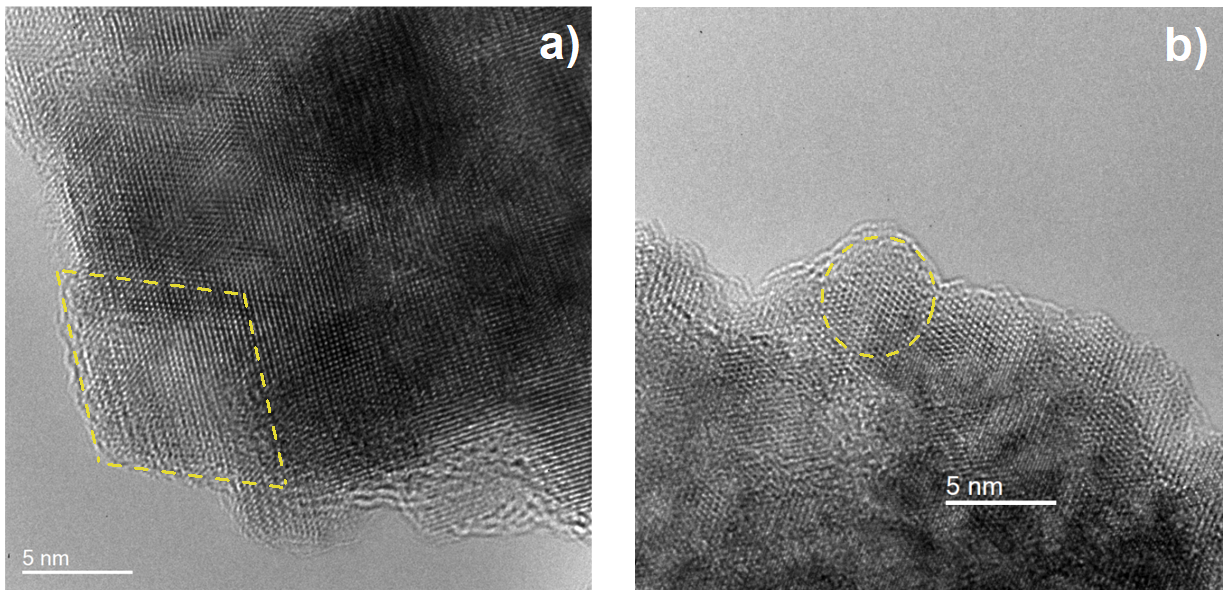


Figure S 9: HR-TEM pictures providing evidence of the crystal alignment beyond the primary crystal size for a) TTEG-a205 and b) TTEG2.

High-angle annular dark field (HAADF) STEM images show dark spots inside the nanoclusters. In this technique, the image contrast is proportional to Z^2^. Black dots in the pictures are related to the presence of interstitial space between CoO primary crystals.

Figure S 10: STEM-HAADF image of TTEG2


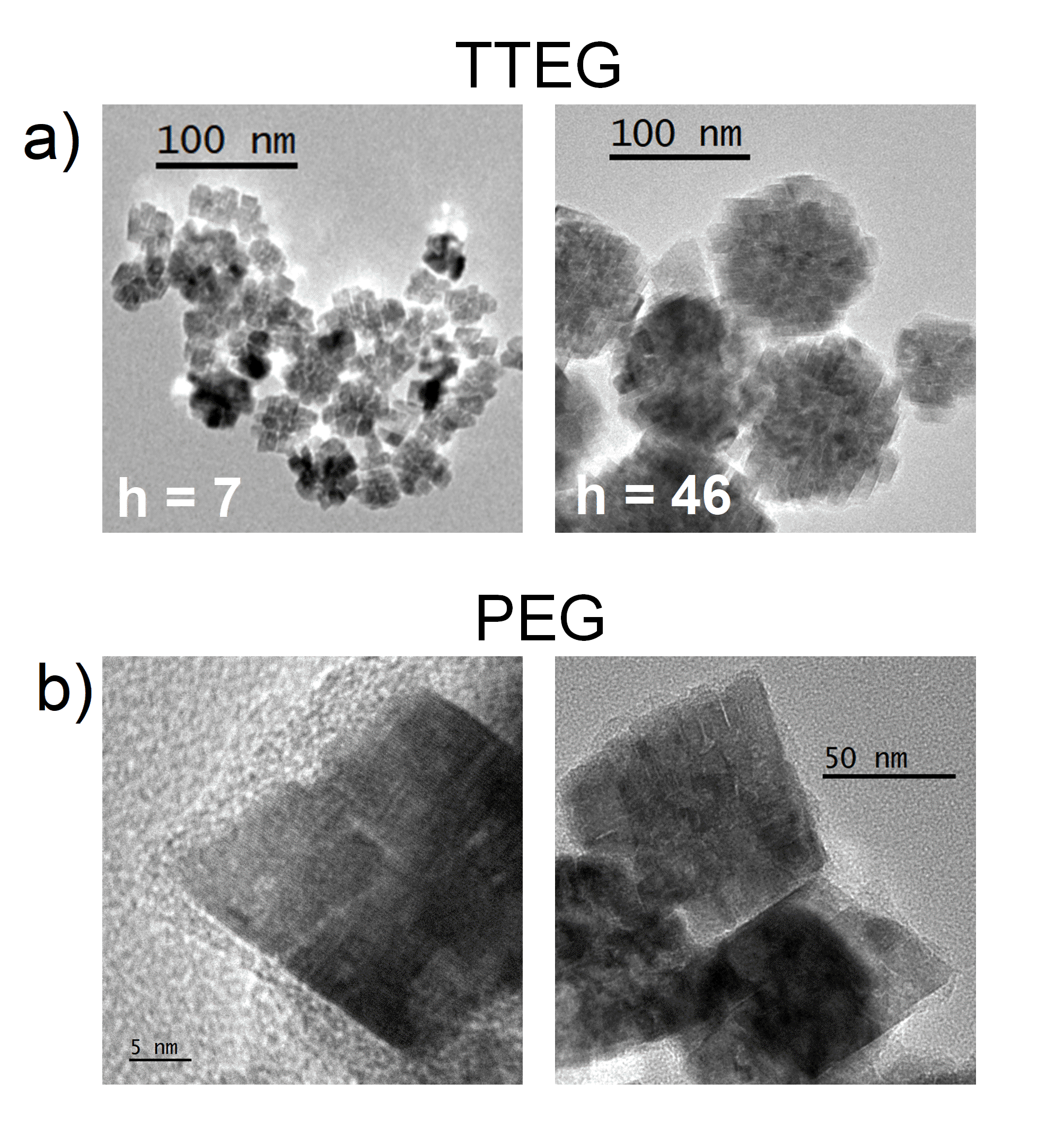


Figure S 11: a panel showing the possible morphological outcomes of the synthesis: a) small nanoflowers and big spherical aggregates in TTEG, with different h, and b) octahedral aggregates (PEG-b235)

The upper and lower branch of the T_N_*vs.*d_XRPD_ graph in the main text (Figure 9) have consistently different morphologies, suggesting that the increased defectiveness of spheroidal NPs based aggregates might have a role in reducing the T_N_ of the nanocrystals, in addition to the mere size effect.


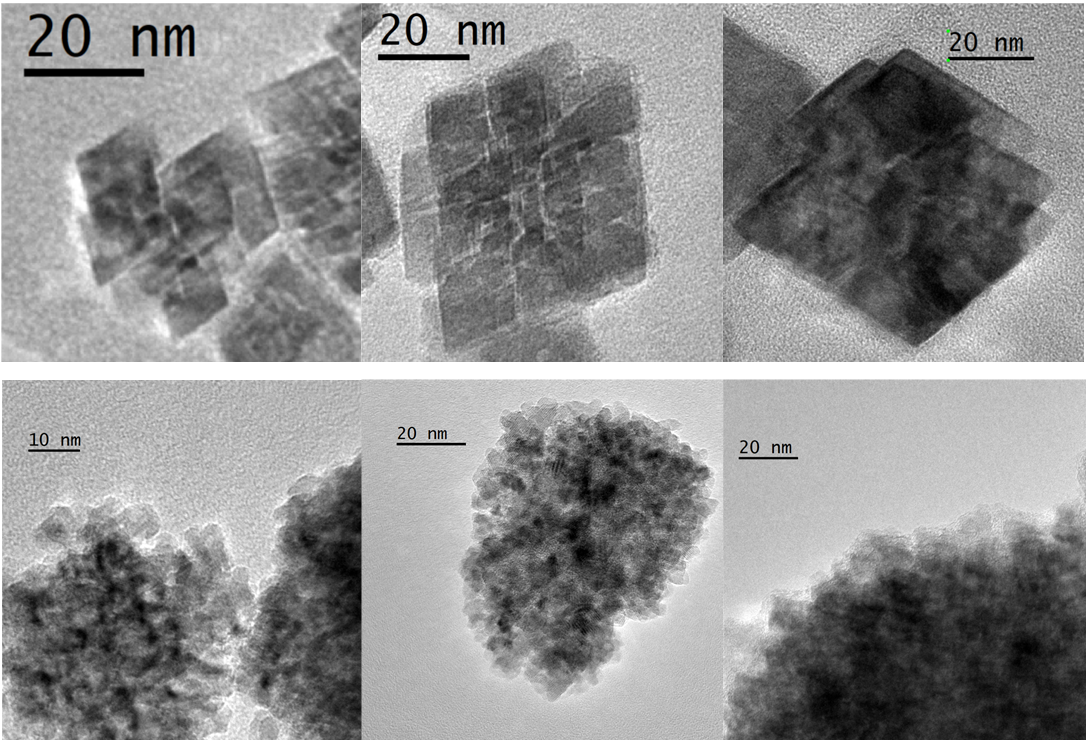


Figure S 12: TEM pictures of samples from the upper branch (top line) and lower branch (bottom line) of Figure 9a in the main text.

# GPA analysis

Geometric Phase Analysis (GPA) was performed to study the stress within the system. The analysis has been carried along the 220 direction of CoO. The stress maps show high percentage values of expansion and contraction, however, due to the profiles of the curves, those values would be given by dislocations within the cluster or rotations between the crystallites and may not be representative of strain value as such. No meaningful distinctions can be made between TTEG2 and TTEG-a205 basing on GPA maps, making difficult to attribute the T_N_ differences to the strain.


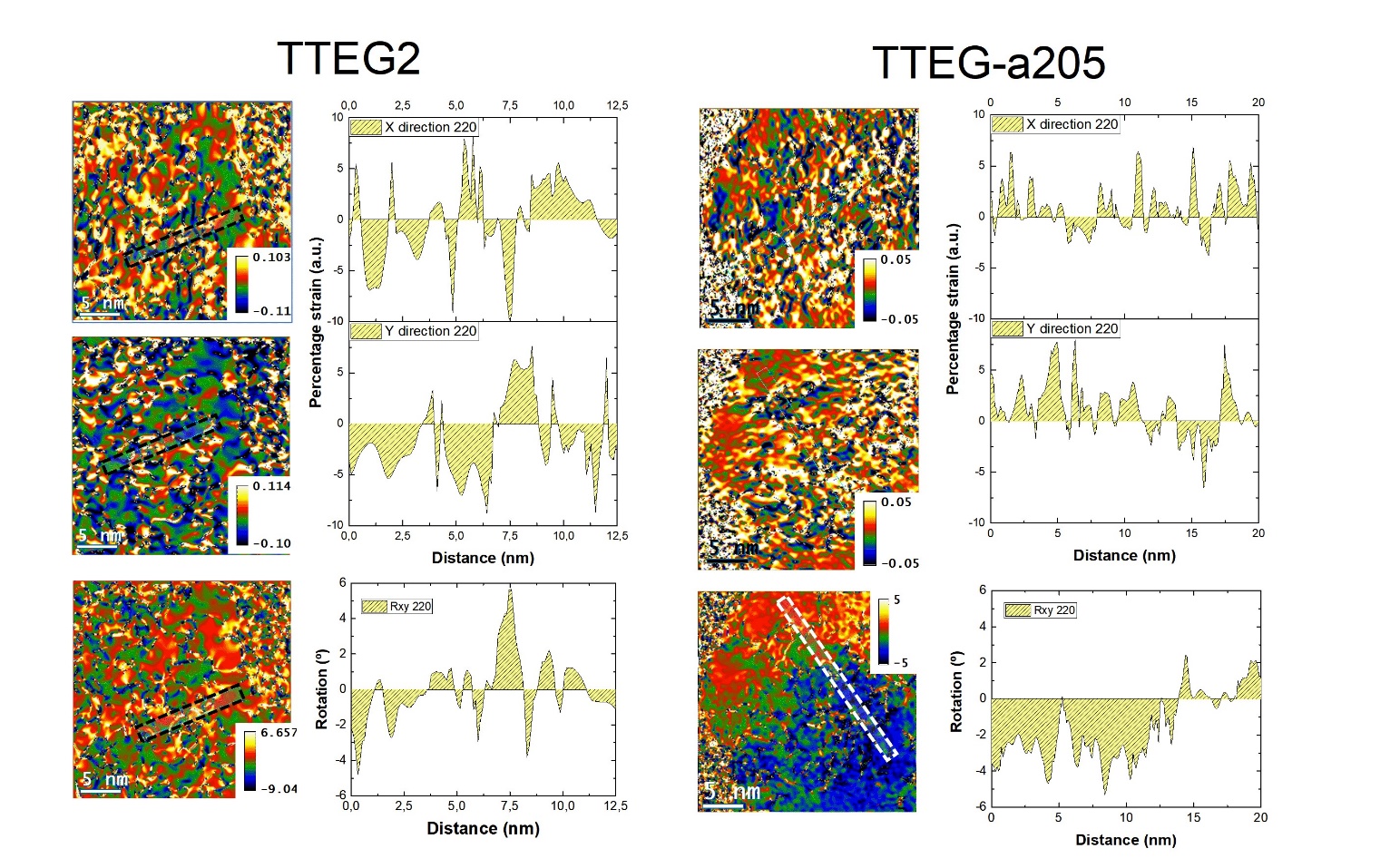


Figure S 13: GPA analyses of TTEG2 and TTEGb1

# Spinel impurities

Electron Energy Loss Spectroscopy (EELS, Figure 5 in main text) is performed to probe the aggregates’ composition. The carbon visible on the surface of the aggregate is due to the polyol residues attached to the material’s surface. A small excess of oxygen is found. The excess is likely owed to aging due to air exposition resulting from the time passed between the synthesis and the HR-TEM characterization.

However, no signs of the presence of spinel are detected with any other technique, especially for the case of the measurements of magnetic properties, which is performed with particularly sensitive tools. Since the magnetic study is always performed on fresh samples, we can conclude that the oxidation happened in the time that passed before the EELS characterization. Furthermore, no Co_3_O_4_ was detected in XRPD patterns of the samples even after HR-TEM experiments, meaning that the maximum quantity possibly developed remains small. For these reasons, the presence of Co_3_O_4_ can be neglected while studying the magnetic properties.


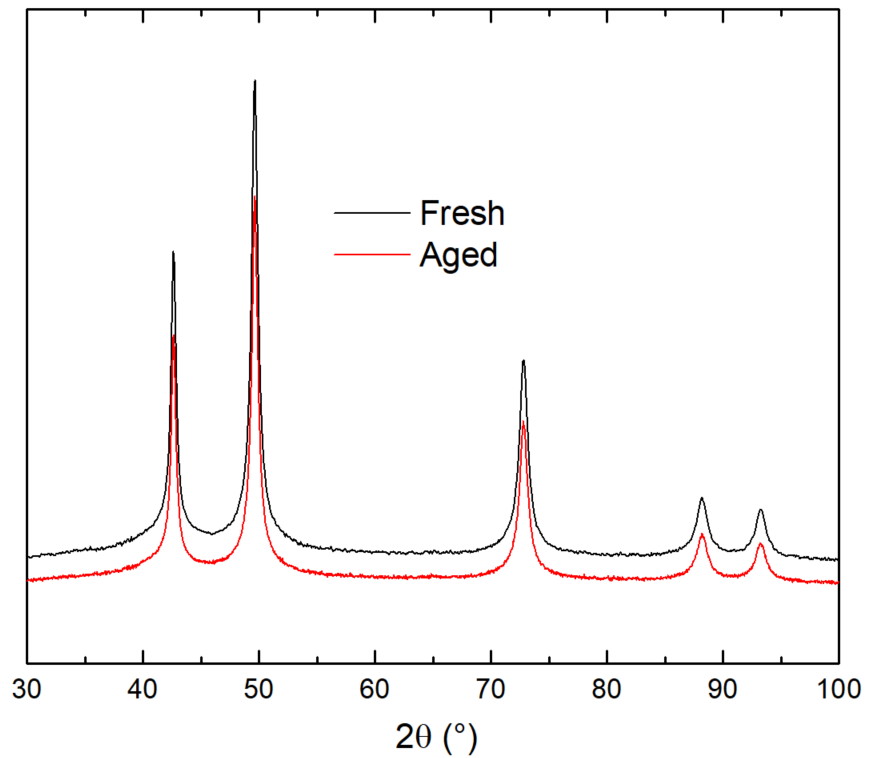


Figure S 14: Comparison of XRPD of fresh and aged TTEG2.

# Magnetic estimation of Co-LHS

The quantity of intermediate in TTEG-b180 is assessed by the weighted sum of the mass-normalized isothermal magnetization plots of a pure CoO sample and the LHS impurity (Int-180), from which its quantity in TTEG-b180 can be estimated to be ~ 3%, explaining why it is not visible in XRD patterns.


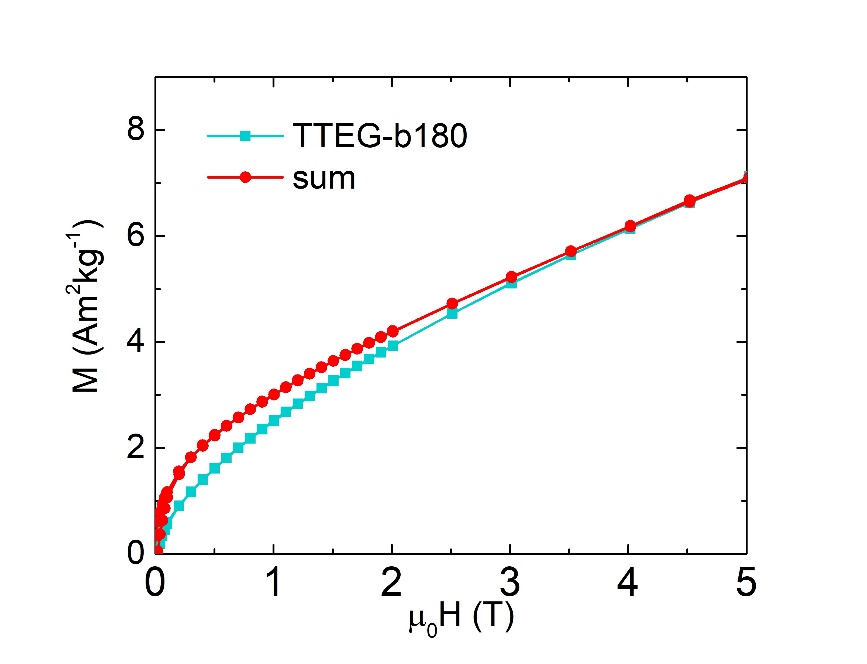


Figure S 16: 2.5 K isothermal magnetization plot of TTEG-b180 and weighted sum of the plots of Int-180 and TTEG2.

# Bibliography

1 L. Poul, S. Ammar-Merah, N. Jouini, F. Fiévet and F. Villain, Metastable solid solutions in the system ZnO-CoO: Synthesis by hydrolysis in polyol medium and study of the morphological characteristics, *Solid State Sci.*, 2001, **3**, 31–42.

2 T. Gaudisson, S. K. Sharma, R. Mohamed, B. S. Youmbi, N. Menguy, F. Calvayrac, M. Seydou and S. Ammar-Merah, Experimental and theoretical evidence for oriented aggregate crystal growth of CoO in a polyol, *CrystEngComm*, 2021, **23**, 1756–1764.
